# Tobacco endgame in the WHO European Region: Feasibility in light of current tobacco control status

**DOI:** 10.18332/tid/174360

**Published:** 2023-11-15

**Authors:** Adrián González-Marrón, Helena Koprivnikar, Judit Tisza, Zsuzsa Cselkó, Angeliki Lambrou, Armando Peruga, Biljana Kilibarda, Cristina Lidón-Moyano, Dolors Carnicer-Pont, Efstathios Papachristou, Emilia Nunes, Giulia Carreras, Giuseppe Gorini, Hipólito Pérez-Martín, Jose M. Martínez-Sánchez, Lorenzo Spizzichino, Maria Karekla, Maurice Mulcahy, Milena Vasic, Otto Ruokolainen, Romain Guignard, Sotiria Schoretsaniti, Tiina Laatikainen, Viêt Nguyen-Thanh, Hanna Ollila

**Affiliations:** 1 Group of Evaluation of Health Determinants and Health Policies, Department of Basic Sciences, Universitat Internacional de Catalunya, Sant Cugat del Vallès, Spain; 2 Department of Health Technology Assessment and Health Economics, Institute for Clinical Effectiveness and Health Policy (IECS), Buenos Aires, Argentina; 3 National Institute of Public Health, Slovenia; 4 National Korányi Institute of Pulmonology, Budapest, Hungary; 5 Directorate of Epidemiology and Prevention of Non-Communicable Diseases and Injuries, National Public Health Organization (NPHO), Athens, Greece; 6 Grupo de Investigación en Control del Tabaco, Institut d'Investigació Biomèdica de Bellvitge (IDIBELL), L'Hospitalet de Llobregat, Barcelona, España; 7 Centro de Investigación Biomédica en Red de Enfermedades Respiratorias, (CIBERES), Madrid, España; 8 Centro de Epidemiología y Políticas de Salud, Facultad de Medicina Clínica Alemana, Universidad del Desarrollo, Santiago, Chile; 9 Institute of Public Health of Serbia “Dr Milan Jovanovic Batut”, Belgrade, Serbia; 10 Programa de Prevenció i Control del Càncer, Institut Català d'Oncologia, L'Hospitalet de Llobregat, Barcelona, España; 11 General Directorate of Health, Ministry of Health, Lisbon, Portugal; 12 Clinical Epidemiology Unit, Oncologic network, prevention and research institute (ISPRO), Florence, Italy; 13 Ministry of Health, Rome, Italy; 14 University of Cyprus, Nicosia, Cyprus; 15 National Environmental Health Service, Health Service Executive (HSE), Galway Business Park, Dangan, Ireland; 16 Faculty of Dentistry, Pancevo, Serbia; 17 Finnish Institute for Health and Welfare, Helsinki, Finland; 18 Santé Publique France, the French National Public Health Agency, Saint-Maurice, France; 19 Institute of Public Health and Clinical Nutrition, University of Eastern Finland, Kuopio, Finland

**Keywords:** advertising and promotion, cessation, end game, packaging and labelling, public policy

## Abstract

**INTRODUCTION:**

To assess the feasibility of developing World Health Organization (WHO) European Region countries’ goals and measures in line with tobacco endgame objectives, information on the current tobacco control context and capacity is needed. The aim of this study was to assess the implementation of the Framework Convention on Tobacco Control (WHO FCTC) and MPOWER measures in the region.

**METHODS:**

In this cross-sectional study we used data from the WHO FCTC implementation reports and MPOWER from 2020 in 53 WHO European Region countries. Six domains (i.e. capacity, taxation and price policies, other national key regulations, public awareness raising and communication, tobacco use cessation, and monitoring) were formed. Subsequently, available indicators under these domains were scored and the level of implementation was computed for each country. Mann-Whitney tests were carried out to compare the scores between the group of countries with and without official endgame goals.

**RESULTS:**

Overall, implementation of the WHO FCTC with the selected indicators at the country level ranged from 28% to 86%, and of MPOWER from 31% to 96%. Full implementation was achieved by 28% of WHO FCTC Parties in the region in taxation and price policies, 12% in public awareness raising and communication, and 42% in monitoring. In capacity, tobacco use cessation and other national key regulations, none of the Parties in the region reached full implementation. Overall median WHO FCTC scores were significantly higher in countries with official endgame goals than in those without (p<0.001).

**CONCLUSIONS:**

There is unequal implementation of both WHO FCTC and MPOWER measures among WHO European Region countries. MPOWER and WHO FCTC provide all the measures for the necessary first steps, followed by innovative measures, to accomplish tobacco endgame goals.

## INTRODUCTION

Tobacco smoking is still one of the leading preventable causes of morbidity and mortality worldwide. In the European Union (EU), around 24% of adult population smoked tobacco products in 2019^1^, and around 0.74 million people die every year due to tobacco smoking^2^. In the World Health Organization (WHO) European Region, the estimated tobacco smoking prevalence in 2015 was 27%^3^ and smoking accounts for 25%, 41% and 63% of cardiovascular disease, cancer and respiratory disease deaths in men, and 6%, 10% and 37% of deaths in women, respectively^4^. To end the tobacco epidemic, the concept of endgame has been put forward. Endgame does not have one definition, but it commonly refers to the situation where the tobacco epidemic is to be ended rather than controlled^5^. Proposed endgame goals and strategies by some countries across the world include reducing the prevalence of tobacco use to a minimal level in the population (<5%) or achieving tobacco-free generations within a specified time frame. In the EU, seven member states already have official endgame goals with differing definitions. These are Belgium, Finland, France, Ireland, the Netherlands, Slovenia, and Sweden. While Ireland^6^ and Sweden^7^ plan to achieve their endgame goal by 2025, Finland^8^, France^9^ and the Netherlands^10^ endgame goals are planned for between 2030 and 2040, and Belgium^11^ and Slovenia^12^ by 2040. Also, in Denmark, an action plan was introduced with a goal on a smoke-free generation by 2030^13^. The EU has also set its overall goal, with the Europe’s Beating Cancer Plan goal to reduce the prevalence of tobacco use in Europe under 5% by 2040^14^. In non-EU countries endgame goals have also been set. Norway aims for a tobacco-free society^15^ and in the UK, England and Scotland aim to be smoke-free by 2030^16^ and 2034, respectively^17^. Importantly, endgame strategies have also been proposed by other organizations (e.g. non-profit organizations)^18^.

To achieve these ultimate tobacco endgame goals, different facilitators have been considered. These include public support for tobacco control in the population, strong political leadership^19^, or going beyond the FCTC mandates implementing innovative measures. However, the first step would be to fully implement the ‘best buys’ defined as part of the MPOWER measures^20^, the WHO Framework Convention on Tobacco Control (FCTC)^21^, and the recommendations in its implementation guidelines^22^. These contain several evidence-based measures, and encourage even going beyond the requirements and recommendations to protect the health of the population^23,24^. In recent years, several innovative measures, such as different market/supply and product-focused measures have been proposed for countries to shift from controlling the tobacco epidemic to ending it. For example, in December 2022, New Zealand adopted a bill which included the ban on the commercial sales of combustible tobacco products to anyone born on or after 1 January 2009, a drastic reduction of around 95% in the number of retailers, and the reduction of nicotine content in cigarettes^25^.

Over the years, the ratification of the WHO FCTC has led to the implementation of key tobacco control measures across several policy domains, which have also resulted in significant reductions of tobacco use^26^. Yet, there is a large variation in the implementation of different WHO FCTC articles and the comprehensiveness and effectiveness of the measures implemented under different articles^27^. In order to inform about the feasibility of achieving tobacco endgame goals and strategies, this study aimed to assess the current status of implementation of key tobacco control measures in WHO European Region countries. Specifically, the aim was to identify potential strengths and deficiencies among countries in the implementation of measures overall and within six domains derived from the WHO FCTC and MPOWER: tobacco control capacity, taxation and price policies, national key regulations, public awareness raising and communication, tobacco use cessation, and monitoring.

## METHODS

### Design

The present study uses cross-sectional data from the WHO FCTC implementation reports and the WHO MPOWER assessments. WHO MPOWER data for the year 2020 were available from the WHO Global Health Observatory^20^. The officially submitted implementation reports of the Parties to the WHO FCTC are publicly available in the WHO FCTC Implementation Database^28^. In the present study, through participation of the WHO FCTC Knowledge Hub on Surveillance, full datasets for the 2020 reporting cycle deriving from the reporting platform of the WHO FCTC, including updated information provided by the Parties, were also utilized. For the countries for which the WHO FCTC implementation report was not available due to recent acceptance of the treaty (i.e. Andorra) or not being part of the Convention (i.e. Monaco and Switzerland), only data from MPOWER were extracted. Hence, WHO FCTC data were utilized for 50 countries while MPOWER data were utilized for 53 countries in the region.

This study was carried out under the project Joint Action on Tobacco Control 2 (JATC2)^29^ Work Package 9 (WP9), which is focused on the best practices to develop an effective and comprehensive tobacco endgame strategy. The WP9 partners representing 15 countries also reviewed their country data and had the possibility to provide recent updates. The updates were minor and did not substantially change the general information gathered from the existing databases.

### Procedure and measures

Indicators assessing the implementation of both WHO FCTC and MPOWER measures were initially identified and extracted by two JATC2 partners from the abovementioned sources of data. These were reviewed and refined by the rest of the partners, and a set of indicators were selected to be included in the current status assessment. The selection process is described in detail in the indicator compendium available on the JATC2 website (www.jaotc.eu).

Given the focus on tobacco endgame, only the strongest level of requirements or recommendations without exemptions were considered from the WHO FCTC and its implementation guidelines. This means that, for example, only advertising bans, not advertising restrictions, were included. For MPOWER, the focus was on established ‘best buys’. Eventually, 106 core indicators (for measures required in the treaty) and 20 advanced indicators (for measures recommended in the treaty or in its implementation guidelines) were included in the current analysis. Implementation was assessed as full if ‘yes’ was reported for ‘yes/no’ questions or ‘complete’ for ‘complete/partial/no’ questions. For indicators with the possible answers ‘complete/partial/no’, partial implementation was determined if ‘partial’ was reported. Non-implementation was determined if ‘no’ was reported for ‘yes/no’ and ‘complete/partial/no’ questions. A score was assigned to each of the indicators, giving two/one point for full, one/half point for partial, and none for no implementation of advanced/core measures, respectively, to weigh for the implementation of advanced measures over core measures in the context of accomplishing an endgame scenario. Details on the grouping and the score of the indicators can be found in the Supplementary file. The overall maximum score was 146 points for all Articles and relevant guidelines.

To provide a better picture of the strengths and challenges in the region, the indicators were further grouped under the following six domains: capacity (i.e. ‘infrastructure’ for tobacco control, strategies, resources, enforcement mechanisms, measures to prevent industry influence and act on industry through liability measures); taxation and price policies (including measures to prevent illicit trade); other national key regulations (i.e. smoking bans applied in indoor settings; testing, measuring and regulation of contents and emissions of tobacco products; packaging and labelling of tobacco products; advertising, promotion and sponsorship; and retail measures to prevent youth access); public awareness raising and communications (i.e. publication of industry data, campaigns, trainings); tobacco use cessation, (i.e. resources directed to cessation of tobacco), and monitoring (i.e. availability of different key data and promoting research). The maximum score for each of the domains was 23 for capacity, 15 for taxation and price policies, 54 for other national key regulations, 13 for public awareness raising and communications, 34 for tobacco use cessation, and 7 for monitoring.

Regarding the MPOWER measures, 10 indicators were used: smoking bans and compliance with smoking bans; health warnings and anti-tobacco mass campaigns; bans in advertising and compliance; offer help to quit smoking; share of total taxes in the retail price, affordability trend (since 2010), % of gross domestic product (GDP) per capita to purchase 2000 cigarettes of the most sold brand. Implementation was assessed using the score reported in the WHO Global Health Observatory^30^. Also, an *ad hoc* score was computed for the indicators ‘affordability trend since 2010 to 2020’, giving 1 point if less affordable in 2020 than in 2010, 0 for no change, and -1 if more affordable in 2020 than in 2010; and ‘% GDP per capita to purchase 2000 cigarettes of the most sold brand’, giving 0 points to countries in the lowest tertile; 0.5 points to countries in the second tertile; and 1 point to countries in the third tertile. These were incorporated into this analysis to provide a better description of the affordability of tobacco at the national level. The overall maximum score was 52 points for MPOWER measures.

### Data analysis

For each country, we estimated the percentage of implementation of the overall WHO FCTC and MPOWER measures, dividing the estimated score by the corresponding maximum. We also computed the percentage of implementation of each WHO FCTC domain per country. Mann-Whitney tests were carried out to compare the scores between the group of countries with and without official endgame goals. Excel version 16.26 and R version 4.2.2, with the package *ggplot2* for graphs, were used for the analyses. The significance level was set at 0.05.

## RESULTS

There were substantial differences in the overall implementation of both WHO FCTC and MPOWER measures among countries, with ranges of 57.6% for WHO FCTC (minimum: 28.4%, maximum: 86.0%) and 65.4% for MPOWER (minimum: 30.8%, maximum: 96.2%), respectively (Table 1).

**Table 1:** Level of implementation of WHO FCTC and MPOWER measures and age-standardized prevalence of tobacco smoking in WHO European Region countries, 2020

*Country*	*WHO FCTC %*	*MPOWER %*	*Age-standardized estimates of current tobacco smoking (2020) %*
Albania	66.4	50.0	22.4
Andorra	NA	48.1	31.8
Armenia	61.3	76.9	25.5
Austria	72.9	86.5	26.4
Azerbaijan	47.3	37.5	20.5
Belarus	74.3	75.0	25.8
Belgium*	75.3	78.8	22.2
Bosnia and Herzegovina	60.3	59.6	35.0
Bulgaria	69.5	73.1	39.0
Croatia	75.0	47.1	36.9
Cyprus	67.5	82.7	35.1
Czech Republic	65.1	89.4	30.7
Denmark	61.3	65.4	17.5
Estonia	60.6	86.5	26.7
Finland*	78.1	89.4	18.2
France*	73.3	87.5	33.4
Georgia	68.2	84.6	31.7
Germany	57.5	67.3	22.0
Greece	63.4	51.0	33.5
Hungary	68.2	48.1	31.8
Iceland	56.8	76.9	12.0
Ireland*	86.3	92.3	20.8
Israel	69.9	41.3	21.2
Italy	65.8	60.6	23.1
Kazakhstan	54.1	67.3	21.1
Kyrgyzstan	76.4	65.4	27.0
Latvia	76.4	87.5	35.0
Lithuania	59.2	68.3	27.4
Luxembourg	63.7	44.2	21.1
Malta	76.7	47.1	24.0
Monaco	NA	30.8	-
Montenegro	62.0	44.2	32.8
The Netherlands*	81.5	65.4	22.2
Norway*	75.3	84.6	16.2
Poland	42.8	77.9	24.0
Portugal	73.3	62.5	25.4
Republic of Moldova	72.6	63.5	25.4
Romania	52.1	78.8	28.0
Russian Federation	67.8	74.0	26.8
San Marino	28.4	42.3	-
Serbia	68.5	69.2	39.8
Slovakia	63.7	68.3	31.5
Slovenia*	80.5	61.5	22.0
Spain	86.0	89.4	27.7
Sweden*	73.6	48.1	15.6
Switzerland	NA	55.8	25.5
Tajikistan	42.1	48.1	-
Republic of North Macedonia	80.8	66.3	-
Turkey	84.6	88.5	30.7
Turkmenistan	85.6	90.4	5.5
Ukraine	39.4	84.6	25.8
United Kingdom*	83.9	96.2	15.4
Uzbekistan	51.0	38.5	10.6

FCTC: Framework Convention on Tobacco Control. NA: not applicable.

* Countries/areas with planned and implemented official tobacco endgame strategies.

Concerning FCTC measures, the lowest percentage of implementation was found in the capacity domain. In the capacity domain, none of the countries reached full implementation. Only 5 out of 50 WHO FCTC parties achieved at least 80% of the maximum score and 33 at least 50% of the maximum score. The percentage of implementation ranged from 17% in Ukraine to 91% in the Netherlands (Figure 1a).

**Figure 1a:**
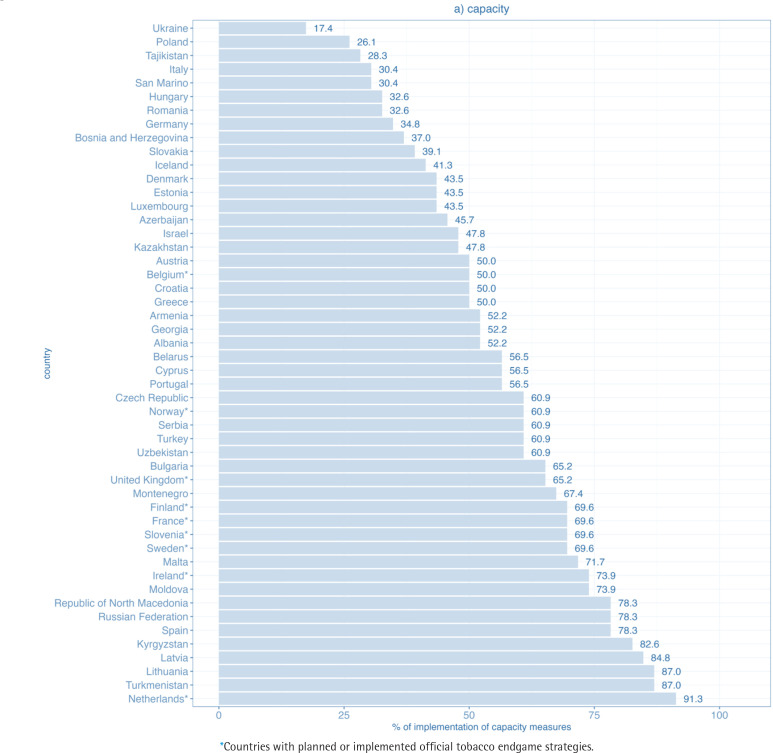
Level of implementation of the domain capacity for WHO FCTC measures in WHO European Region countries (2020)

Within the taxation and price policies domain, 30 out of 50 WHO European Region FCTC parties achieved at least 80% of the maximum score, and 47 at least 50% of the maximum score. Altogether 14 WHO FCTC parties reported full implementation. Lowest percentage of implementation was 21% (San Marino) (Figure 1b).

**Figure 1b:**
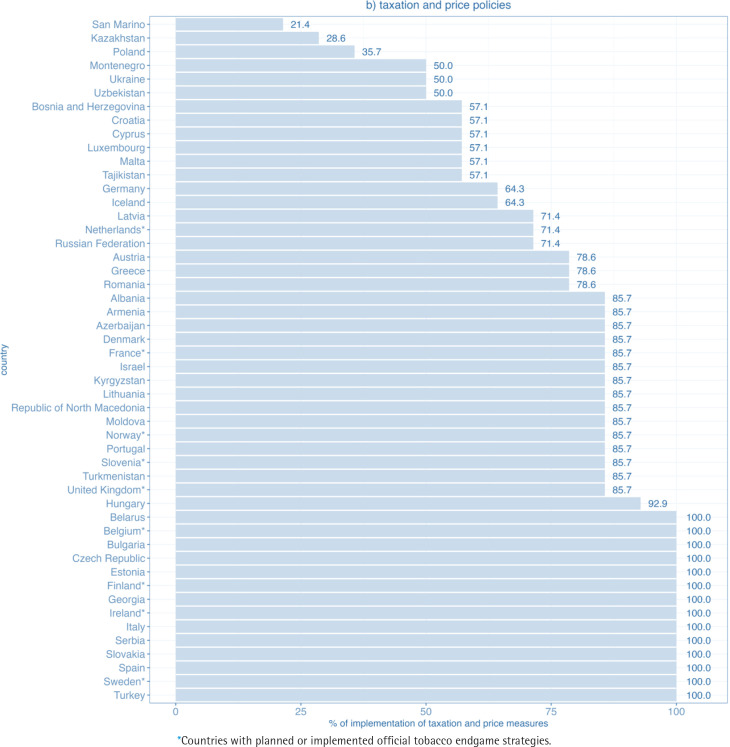
Level of implementation of the domain taxation and price policies for WHO FCTC measures in WHO European Region countries (2020)

The second domain with the highest implementation was other national key regulations. Among 50 WHO European Region FCTC parties, 27 achieved 80% or more of the maximum score, while almost all, that is 47, achieved at least 50% of the maximum score. The percentage of implementation ranged from 25% in Poland to 99% in Slovenia (Figure 1c). Still, none of the Parties in the region reported full implementation.

**Figure 1c:**
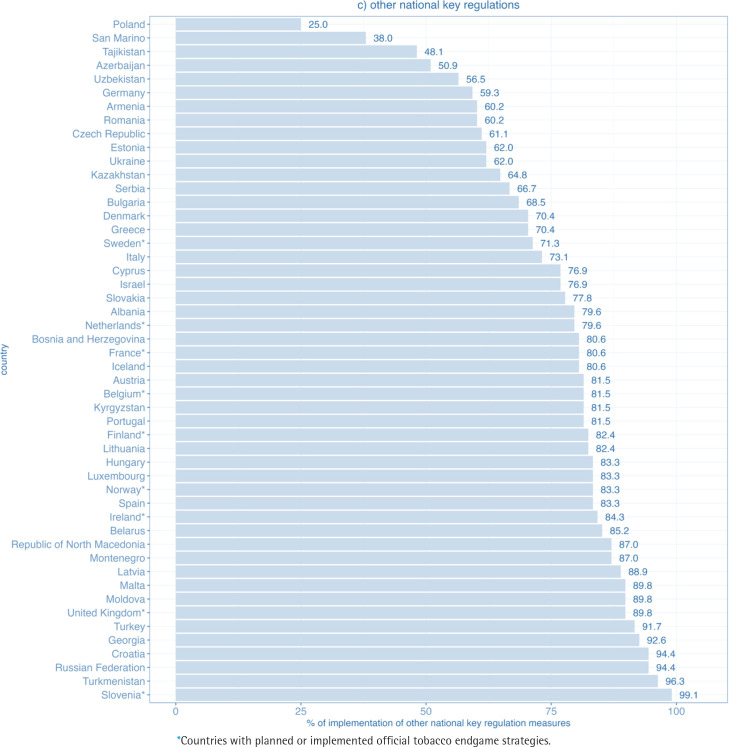
Level of implementation of the domain other national key regulations for WHO FCTC measures in WHO European Region countries (2020)

In the public awareness raising and communications domain, 19 out of 50 WHO European Region FCTC parties achieved at least 80% of the maximum score and 39 at least 50% of the maximum score. Altogether 6 Parties reported full implementation. The lowest percentage of implementation was 15% (Denmark) (Figure 1d).

**Figure 1d:**
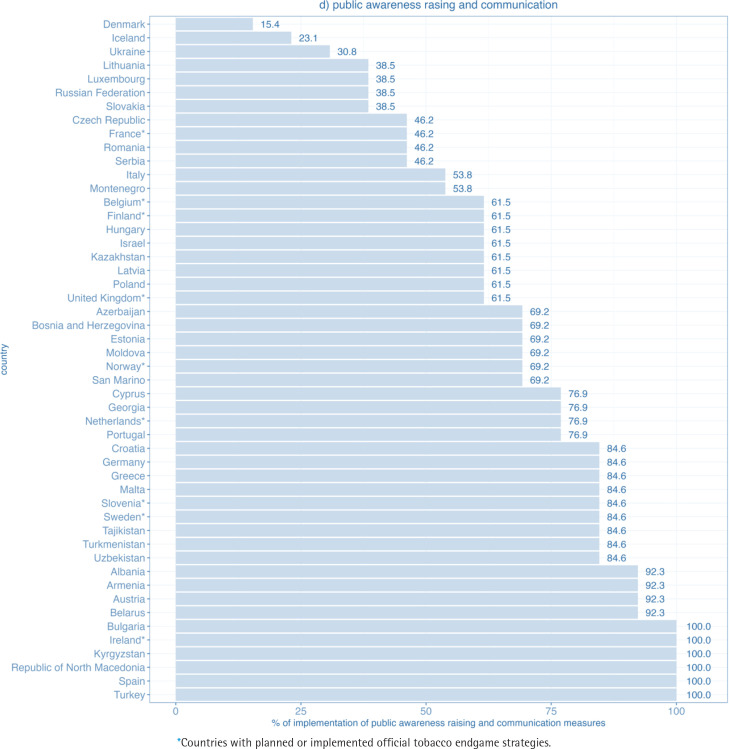
Level of implementation of the domain public awareness raising and communication for WHO FCTC measures in WHO European Region countries (2020)

In the tobacco use cessation domain, only 3 out of 50 WHO FCTC parties achieved at least 80% of the maximum score and 31 at least 50% of the maximum score. The percentage of implementation ranged from 6% in San Marino to 91% in the United Kingdom (Figure 1e). None of the Parties in the region reached full implementation.

**Figure 1e:**
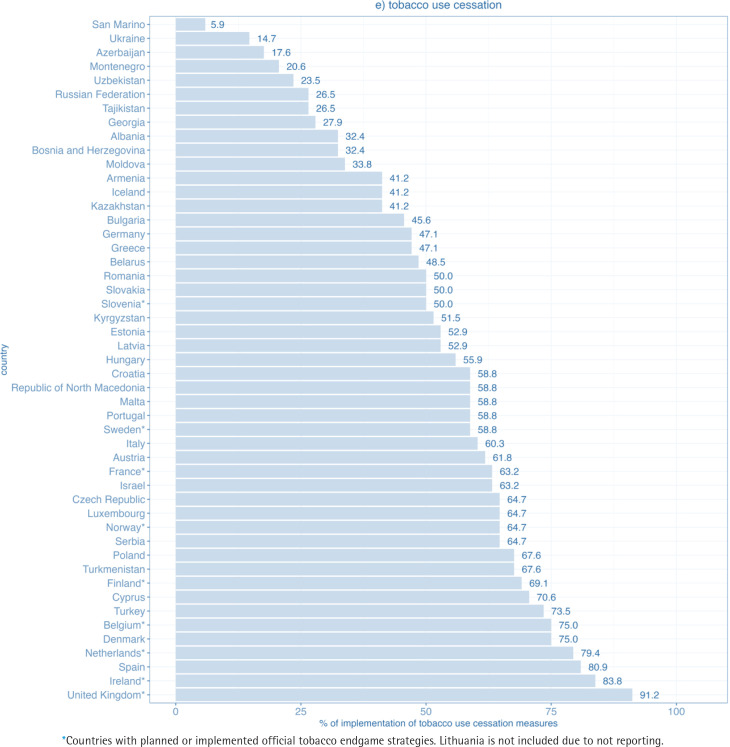
Level of implementation of the domain tobacco use cessation for WHO FCTC measures in WHO European Region countries (2020)

The highest percentage of implementation was found in the monitoring domain, where among the 50 WHO European Region FCTC parties, 21 achieved full implementation, 32 achieved 80% or more of the maximum score, and 44 achieved at least 50% of the maximum score (Figure 1f).

**Figure 1f:**
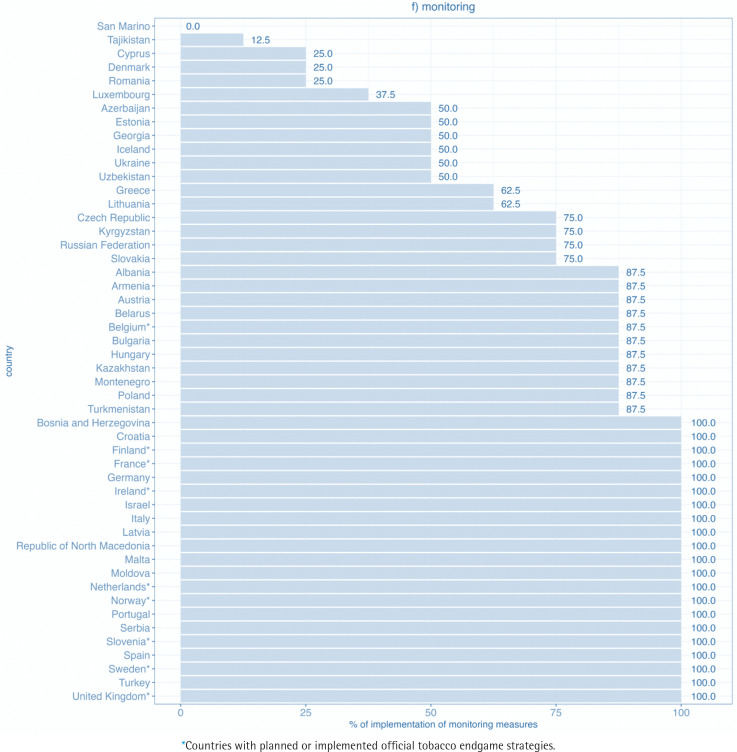
Level of implementation of the domain monitoring for WHO FCTC measures in WHO European Region countries (2020)

There were significant differences in the median overall WHO FCTC scores in countries with [78.1 (IQR: 75.3–81.5)] and without [65.8 (IQR: 59.2– 72.9)] official endgame goals (p<0.001). Significant differences were also found in the capacity, tobacco use cessation, and monitoring domains (Table 2).

**Table 2:** Median (and interquartile range) MPOWER and WHO FCTC scores, overall and according to domains, 2020

	*Countries without official endgame goals (N=41) Median (IQR)*	*Countries with official endgame goals (N=9) Median (IQR)*	p*
**Overall WHO FCTC**	65.8 (59.2–72.9)	78.1 (75.3–81.5)	<0.001
**WHO FCTC domains**			
Capacity	52.2 (41.3–65.2)	69.6 (65.2–69.6)	0.017
Taxation and price policies	85.7 (57.1–92.9)	85.7 (85.7–100.0)	0.062
Other national key regulations	79.6 (62.0–85.2)	82.4 (80.6–84.3)	0.136
Public awareness raising and communication	69.2 (53.8–84.6)	69.2 (61.5–84.6)	0.889
Tobacco use cessation	50.8 (33.5–62.2)	69.1 (63.2–79.4)	0.002
Monitoring	87.5 (50.0–100)	100.0 (100.0–100.0)	0.003
**MPOWER**	66.8 (48.1–78.1)	84.6 (65.4–89.4)	0.037

IQR: interquartile range. WHO FCTC: World Health Organization Framework Convention on Tobacco Control.

* Mann-Whitney test.

## DISCUSSION

We have observed wide differences between WHO European Region countries in the implementation of both WHO FCTC and MPOWER measures, meaning there is plenty of room for improvement in maximizing the implementation of measures of WHO FCTC and its implementation guidelines. Importantly, although the implementation of different tobacco control policies has been assessed already through diverse tools^31^ and in different reports^32^, this is, as far as we know, the first study assessing the implementation of tobacco control measures at their strongest level, which would be required to achieve tobacco endgame goals.

In all six domains (i.e. capacity, monitoring, other national key regulations, public awareness raising and communication, taxation and prices, and tobacco use cessation), notable differences between countries were also observed. Considering each of the domains, the highest level of implementation of measures of WHO FCTC and associated guidelines is in the monitoring domain, for which many parties achieve full implementation. This means that monitoring systems are well established in a great part of the countries, including data availability on smoking prevalence and other relevant indicators for argumentation of new measures or evaluation of implemented ones. Secondly, an estimated 54% of parties have implemented at least 80% of measures in the other national key regulations domain. However, the WHO FCTC implementation database does not include indicators describing the level of compliance with the measures, which would show the actual implementation of those measures in practice. Thirdly, and surprisingly, 60% of the parties have implemented at least 80% of the measures within the taxation and prices domain, which also included measures to control illicit trade in the current assessment. Based on the WHO data on prices of cigarettes in international dollars at purchasing power parity^30^, we would assume that a lower percentage of countries would show high implementation. In this case, we believe that some other indicators would benefit the analysis to show the actual differences in the taxation and pricing of tobacco products. In Europe, the EU-wide tracking and tracing regime may also explain some of the higher scores in the current analysis. Regarding the public awareness raising and communications domain, 38% of the parties achieved at least 80% implementation. Here we assume that the most extensive differences among WHO FCTC parties reporting are present fundamentally on awareness and training programs in different settings, due to no validation of reported data in the WHO FCTC Implementation Database. Finally, the lowest percentages of implementation were found in the capacity and smoking cessation domains, with only 10% and 6% of parties having implemented at least 80% of the measures, respectively. Public funding or reimbursement schemes are essential in order to achieve a higher rate of implementation of WHO FCTC Article 14 measures.

Importantly, there are relevant within-country variations in the implementation of measures included in different domains. As a result, no country at the moment is among the top ten in the implementation of measures across all six domains. Ireland is in the top ten in 5 out of the 6 domains, and Latvia, Finland, Turkey and Spain top ten in 3 of the 6 domains. These results, except for Latvia, are similar to those observed in the Tobacco Control Scale 2021^33^, in which Ireland is in the top and Finland, Turkey and Spain within the first tertile of the scale. Nevertheless, a higher ranking in these 5 countries is not necessarily reflected in lower smoking prevalence, a fact which is also true for countries with the highest achieved percentage of the overall implementation of WHO FCTC measures or MPOWER measures, showing there is still plenty of room to reduce the prevalence of tobacco smoking in Europe^34^. This is also supported by the WHO projections on reaching the 30% relative reduction in tobacco use prevalence between 2010 and 2025, as set in the Global Action Plan on non-communicable diseases (NCD). Based on the 2021 assessment, the WHO European Region was seeing a relatively slow rate of decline, currently tracking towards a 19% relative reduction between 2010 and 2025^3^. Reaching this NCD goal is the interim target of the Tobacco-Free Generation goal in the EU Cancer Plan^14^, making it even more important to strengthen the implementation of key tobacco control measures. Given this, and since there is evidence that the prevalence of tobacco use is mainly reduced when national tobacco policies are comprehensively implemented, and also that synergistic effects are observed when different policies are implemented simultaneously^35^, further simultaneous tobacco control efforts should be taken in individual countries. It is also important to point out that discrepancies between ranks and prevalence of smoking might be due to variations in the level of compliance (which is not assessed in our analysis), higher impact of some measures depending on the country context, sociocultural differences among countries and also due to the differences in methodologies used for obtaining data on tobacco use prevalence, that are further used by WHO for producing estimates and standardized smoking rates. Simulation modelling studies, as carried out elsewhere^36^, should be realized at the WHO European Region level to fully understand the association between the different endgame facilitators put forward (e.g. public support for tobacco control in the population, strong political leadership)^19^ and the probability of achieving tobacco endgame goals.

Significant differences were found in the implementation of WHO FCTC measures between countries with and without official endgame strategies regarding, capacity, tobacco use cessation, and monitoring. Still, the countries that established official tobacco endgame goals have not implemented all the key requirements and recommendations from the WHO FCTC or MPOWER. These results warrant attention and action in these countries, as the effectiveness of innovative tobacco endgame measures can be undermined by the lack of implementation of key evidence-based measures.

Overall, and according to our results, the feasibility of accomplishing supranational endgame goals in Europe, such as the one proposed in the Europe’s Beating Cancer Plan of reducing the prevalence of tobacco use in Europe under 5% by 2040^14^, may be hampered by the low implementation of WHO FCTC and MPOWER measures in a number of countries. However, at the same time, establishing national tobacco endgame goals can provide the opportunity to bring the need for strengthened implementation of the WHO FCTC and MPOWER to the political agenda as part of the national measures for achieving the goal.

### Strengths and limitations

Our study should be interpreted considering some limitations. Firstly, the WHO FCTC implementation reports are completed by national focal points and did not go through a validation process. In this sense, inter-reporter validity may be low, which may bias comparisons between countries and between WHO FCTC and MPOWER assessment. For example, WHO FCTC party reporting on smoking bans may indicate complete protection, even though smoking cabins are allowed in certain enclosed places. However, in MPOWER, such a situation is assessed as incomplete protection.

Also, the lack of available evidence in the WHO FCTC indicators on compliance with tobacco control measures, missing data in MPOWER indicators regarding compliance with smoke-free spaces and with bans on advertising, and the different indicators used for assessing taxation between MPOWER and WHO FCTC, may be the reason for the differences between the estimation obtained for both groups of measures.

Moreover, qualitative data from the WHO FCTC Implementation Database, which may have added further context to the implementation status, were not considered in our analysis. Also, while the majority of indicators retained from MPOWER had a validated score, which we obtained from the WHO Global Health Observatory^20^, scores for ‘affordability trend since 2010 to 2020’ and ‘% GDP per capita to purchase 2000 cigarettes of the most sold brand’ were estimated *ad hoc* and may not correctly estimate the implementation of these measures. Besides, implementation of the indicators was assessed using a semi-quantitative scoring (i.e. scores were assigned to the categories of implementation), which lack the discrimination capacity of continuous scales.

The update of the status of implementation of the WHO FCTC indicators at the national level performed by JATC2 WP9 partners, resulted in only a few updates which requested only minor corrections of the data gathered from WHO FCTC reports. This indicated the data gathered are complete and updated.

Finally, our study is the first carried out to estimate the implementation of both WHO FCTC and MPOWER measures in WHO European Region countries in the context of tobacco endgame, a relatively new field in tobacco control. For this reason, this study may be considered a road map to identify the gaps to achieve the objective in the Europe’s Beating Cancer Plan of reducing the prevalence of tobacco smoking under 5% by 2040.

## CONCLUSIONS

There are wide differences in the implementation of both WHO FCTC and MPOWER measures among WHO European Region countries. Further tobacco control regulations in order to achieve full implementation of core and advanced WHO FCTC measures are needed, especially in the capacity and smoking cessation domains, to accomplish the Europe’s Beating Cancer Plan goal of Tobacco-free Generation.

## Data Availability

The data supporting this research can be found in the Supplementary file.
