# Supplementary material for: Tobacco endgame in the WHO European Region: Feasibility in light of current tobacco control status
Source: Tob Induc Dis. 2023 Nov 15;21:151. doi: 10.18332/tid/174360 (PMC10647070; doi:10.18332/tid/174360)
Supplement: Supplementary file 1 [file TID-21-151-s1.pdf]

	Capacity																
	Have you developed and implemented comprehensive multisectoral national tobacco control strategies, plans and programmes in accordance with the Convention?	Have you partially developed and implemented tobacco control strategies by including tobacco control in national health, public health or health promotion strategies, plans and programmes?	score_strategies_core	Have you established or reinforced and financed: [a focal point for tobacco control?]	score_focal_core	Have you established or reinforced and financed: [a tobacco control unit?]	score_tcunit_core	Have you established or reinforced and financed: [a national coordinating mechanism for tobacco control?]	score_natcoord_core	protecting public health policies with respect to tobacco control from commercial and other vested interests of the tobacco industry?	score_commercial_core	Do you earmark any percentage of your taxation income for funding any national plan or strategy on tobacco control in your jurisdiction?	score_earmark_adv	Do any of these measures provide for a mechanism/ infrastructure for enforcement?	score_enforcement_adv	awareness and participation of the following agencies and organizations in development and implementation of intersectoral programmes and strategies for tobacco control: [public agencies?]	score_publicagencies_core
Albania	Yes	N/A	1	Yes	1	Yes	1	Yes	1	Yes	1	No	0	Yes	1	Yes	1
Andorra																	
Armenia	Yes	N/A	1	Yes	1	No	0	Yes	1	No	0	No	0	N/A	0	Yes	1
Austria	No	Yes	0.5	Yes	1	Yes	1	No	0	Yes	1	No	0	Yes	1	Yes	1
Azerbaijan	No	No	0.5	Yes	1	No	0	No	0	Yes	1	No	0	Yes	1	Yes	1
Belarus	Yes	N/A	1	No	0	Yes	1	Yes	1	Yes	1	No	0	Yes	1	Yes	1
Belgium	No	Yes	0.5	Yes	1	Yes	1	Yes	1	No	0	N/A	0	Yes	1	Yes	1
Bosnia and Herzegovina	No	Yes	0.5	No	0	Yes	1	No	0	No	0	No	0	Yes	1	Yes	1
Bulgaria	Yes	N/A	1	Yes	1	Yes	1	No	0	Yes	1	N/A	0	Yes	1	Yes	1
Croatia	No	Yes	0.5	Yes	1	Yes	1	Yes	1	Yes	1	No	0	No	0	Yes	1
Cyprus	Yes	N/A	1	Yes	1	Yes	1	Yes	1	Yes	1	No	0	Yes	1	Yes	1
Czech Republic	Yes	N/A	1	Yes	1	No	0	Yes	1	Yes	1	No	0	Yes	1	Yes	1
Denmark	Yes	N/A	1	Yes	1	Yes	1	Yes	1	Yes	1	No	0	Yes	1	No	0
Estonia	Yes	N/A	1	Yes	1	Yes	1	Yes	1	No	0	No	0	No	0	Yes	1
Finland	Yes	N/A	1	Yes	1	Yes	1	No	0	Yes	1	No	0	Yes	1	Yes	1
France	Yes	N/A	1	No	0	No	0	Yes	1	Yes	1	Yes	1	Yes	1	Yes	1
Georgia	Yes	N/A	1	Yes	1	Yes	1	Yes	1	Yes	1	No	0	Yes	1	Yes	1
Germany	Yes	N/A	1	Yes	1	Yes	1	Yes	1	No	0	No	0	Yes	1	Yes	1
Greece	No	Yes	0.5	Yes	1	No	0	Yes	1	No	0	No	0	Yes	1	Yes	1
Hungary	No	Yes	0.5	Yes	1	Yes	1	Yes	1	Yes	1	No	0	Yes	1	Yes	1
Iceland	No	Yes	0.5	Yes	1	Yes	1	Yes	1	Yes	1	Yes	1	Yes	1	No	0
Ireland	Yes	N/A	1	Yes	1	Yes	1	Yes	1	Yes	1	Yes	1	Yes	1	Yes	1
Israel	Yes	N/A	1	Yes	1	No	0	Yes	1	Yes	1	No	0	Yes	1	Yes	1
Italy	Yes	N/A	1	Yes	1	Yes	1	No	0	No	0	No	0	Yes	1	Yes	1
Kazakhstan	Yes	N/A	1	No	0	No	0	No	0	Yes	1	No	0	Yes	1	Yes	1
Kyrgyzstan	Yes	N/A	1	Yes	1	Yes	1	Yes	1	Yes	1	No	0	Yes	1	Yes	1
Latvia	No	Yes	0.5	Yes	1	Yes	1	Yes	1	No	0	No	0	Yes	1	Yes	1
Lithuania	Yes	N/A	1	Yes	1	Yes	1	Yes	1	Yes	1	Yes	1	Yes	1	Yes	1
Luxembourg	Yes	N/A	1	No	0	No	0	Yes	1	Yes	1	No	0	Yes	1	Yes	1
Malta	No	Yes	0.5	Yes	1	Yes	1	Yes	1	Yes	1	No	0	Yes	1	Yes	1
Monaco																	
Montenegro	No	Yes	0.5	Yes	1	Yes	1	Yes	1	No	0	No	0	Yes	1	Yes	1
Netherlands	Yes	N/A	1	Yes	1	Yes	1	Yes	1	Yes	1	No	0	Yes	1	Yes	1
Norway	Yes	N/A	1	Yes	1	Yes	1	No	0	No	0	No	0	Yes	1	Yes	1
Poland	Yes	N/A	1	Yes	1	Yes	1	No	0	Yes	1	No	0	N/A	0	Yes	1
Portugal	Yes	N/A	1	Yes	1	Yes	1	Yes	1	Yes	1	No	0	Yes	1	Yes	1
Republic of Moldova	Yes	N/A	1	Yes	1	Yes	1	Yes	1	Yes	1	No	0	Yes	1	Yes	1
Romania	No	Yes	0.5	Yes	1	No	0	No	0	Yes	1	Yes	1	Yes	1	Yes	1
Russian Federation	Yes	N/A	1	No	0	No	0	Yes	1	Yes	1	No	0	Yes	1	Yes	1
San Marino	Yes	N/A	1	Yes	1	No	0	No	0	Yes	1	No	0	Yes	1	Yes	1
Serbia	Yes	N/A	1	Yes	1	Yes	1	No	0	Yes	1	No	0	Yes	1	Yes	1
Slovakia	Yes	N/A	1	Yes	1	No	0	Yes	1	Yes	1	No	0	Yes	1	Yes	1
Slovenia	Yes	Yes	1	Yes	1	Yes	1	Yes	1	Yes	1	No	0	Yes	1	Yes	1
Spain	Yes	N/A	1	Yes	1	Yes	1	Yes	1	Yes	1	No	0	Yes	1	Yes	1
Sweden	Yes	N/A	1	Yes	1	Yes	1	Yes	1	No	0	No	0	Yes	1	Yes	1
Switzerland																	
Tajikistan	No	Yes	0.5	No	0	No	0	No	0	Yes	1	No	0	No	0	Yes	1
The former Yugoslav Republic of	Yes	N/A	1	Yes	1	No	0	No	0	Yes	1	N/A	0	Yes	1	Yes	1
Turkey	Yes	N/A	1	Yes	1	Yes	1	Yes	1	Yes	1	No	0	Yes	1	Yes	1
Turkmenistan	Yes	N/A	1	Yes	1	No	0	Yes	1	Yes	1	No	0	Yes	1	Yes	1
Ukraine	No	No	0	No	0	No	0	No	0	Yes	1	No	0	Yes	1	No	0
United Kingdom	Yes	N/A	1	Yes	1	Yes	1	Yes	1	Yes	1	No	0	Yes	1	Yes	1
Uzbekistan	Yes	N/A	1	Yes	1	No	0	Yes	1	Yes	1	No	0	Yes	1	Yes	1

	Capacity															
	awareness and participation of the following agencies and organizations in development and implementation of intersectoral programmes and strategies for tobacco control: [nongovernmental organizations not affiliated with the tobacco industry?]	score_nongov_core	awareness and participation of the following agencies and organizations in development and implementation of intersectoral programmes and strategies for tobacco control: [private organizations?]	score_privorg_core	Is there any tobacco-growing in your jurisdiction?	score_growing_core	Does your tobacco control legislation contain measures regarding criminal liability for any violations of that tobacco control legislation? []	score_crimtc_adv	Do you have separate criminal liability provisions in relation to tobacco control (outside of the tobacco control legislation)? []	score_crimouts_adv	Do you have any civil liability measures that are specific to tobacco control? []	score_civspe_adv	Do you have any general civil liability provisions that could apply to tobacco control? []	score_civany_adv	Do you have civil or criminal liability provisions that provide for compensation for adverse health effects and/or for reimbursement of medical, social or other relevant costs? []	score_advefcom_adv
Albania	Yes	1	Yes	1	Yes	0	Yes	1	No	0	No	0	No	0	No	0
Andorra																
Armenia	Yes	1	Yes	1	Yes	0	No	0	Yes	1	No	0	Yes	1	Yes	1
Austria	Yes	1	Yes	1	No	1	No	0	Yes	1	No	0	No	0	No	0
Azerbaijan	Yes	1	No	0	Yes	0	No	0	No	0	Yes	1	No	0	Yes	1
Belarus	Yes	1	No	0	No	1	No	0	No	0	Yes	1	Yes	1	No	0
Belgium	Yes	1	No	0	Yes	0	Yes	1	No	0	No	0	Yes	1	No	0
Bosnia and Herzegovina	No	0	No	0	Yes	0	Not applica	0	Yes	1	Yes	1	Not applica	0	Not applicabl	0
Bulgaria	Yes	1	Yes	1	Yes	0	Yes	1	Yes	1	No	0	Yes	1	Not applicabl	0
Croatia	Yes	1	Yes	1	Yes	0	Yes	1	Yes	1	No	0	No	0	No	0
Cyprus	Yes	1	Yes	1	No	1	Yes	1	Not applica	0	No	0	Not applica	0	No	0
Czech Republic	Yes	1	Yes	1	No	1	No	0	Yes	1	No	0	Yes	1	No	0
Denmark	No	0	No	0	No	1	Yes	1	No	0	Not applica	0	Not applica	0	No	0
Estonia	No	0	No	0	No	1	Yes	1	Yes	1	No	0	No	0	No	0
Finland	Yes	1	Yes	1	No	1	Yes	1	No	0	No	0	Yes	1	Yes	1
France	Yes	1	Yes	1	Yes	0	Yes	1	Yes	1	No	0	Yes	1	No	0
Georgia	Yes	1	Yes	1	Yes	0	No	0	Yes	1	No	0	No	0	No	0
Germany	Yes	1	No	0	Yes	0	No	0	No	0	No	0	No	0	No	0
Greece	Yes	1	Yes	1	Yes	0	Yes	1	No	0	No	0	Yes	1	No	0
Hungary	No	0	No	0	Yes	0	No	0	No	0	No	0	No	0	Not applicabl	0
Iceland	No	0	No	0	No	1	No	0	No	0	No	0	No	0	No	0
Ireland	Yes	1	Yes	1	No	1	Yes	1	Yes	1	No	0	No	0	No	0
Israel	Yes	1	No	0	No	1	Yes	1	No	0	No	0	No	0	No	0
Italy	Yes	1	No	0	Yes	0	No	0	No	0	No	0	No	0	No	0
Kazakhstan	Yes	1	No	0	No	1	No	0	No	0	Yes	1	Yes	1		0
Kyrgyzstan	Yes	1	No	0	Yes	0	Yes	1	Yes	1	Yes	1	Yes	1	Yes	1
Latvia	Yes	1	Yes	1	No	1	Yes	1	Yes	1	Yes	1	Yes	1	Yes	1
Lithuania	Yes	1	No	0	No	1	Yes	1	Yes	1	Yes	1	Yes	1	Not applicabl	0
Luxembourg	Yes	1	No	0	No	1	Yes	1	No	0	No	0	No	0	No	0
Malta	Yes	1	Yes	1	No	1	Yes	1	No	0	No	0	Yes	1	Yes	1
Monaco																
Montenegro	Yes	1	No	0	Yes	0	Yes	1	Yes	1	Yes	1	Yes	1	No	0
Netherlands	Yes	1	Yes	1	No	1	Yes	1	Yes	1	Yes	1	Yes	1	Yes	1
Norway	Yes	1	No	0	No	1	Yes	1	No	0	No	0	Yes	1	Yes	1
Poland	Yes	1	No	0	Yes	0	No	0	No	0	Not applica	0	Not applica	0	No	0
Portugal	Yes	1	No	0	Yes	0	No	0	No	0	Yes	1	Yes	1	No	0
Republic of Moldova	Yes	1	No	0	Yes	0	Yes	1	Not applica	0	Yes	1	Yes	1	Yes	1
Romania	No	0	No	0	Yes	0	No	0	No	0	No	0	No	0	No	0
Russian Federation	Yes	1	Yes	1	Yes	0	Yes	1	Yes	1	Yes	1	Yes	1	Yes	1
San Marino	No	0	No	0	No	1	Not applica	0	Not applica	0	Not applica	0	Not applica	0	Not applicabl	0
Serbia	Yes	1	No	0	Yes	0	No	0	Yes	1	No	0	Yes	1	Yes	1
Slovakia	No	0	No	0	Yes	0	No	0	No	0	No	0	Yes	1	No	0
Slovenia	Yes	1	No	0	No	1	Yes	1	No	0	No	0	Yes	1	Yes	1
Spain	Yes	1	Yes	1	Yes	0	Yes	1	Yes	1	Yes	1	Yes	1	No	0
Sweden	Yes	1	Yes	1	No	1	Yes	1	Yes	1	No	0	Yes	1	No	0
Switzerland																
Tajikistan	No	0	Yes	1	No	1	No	0	No	0	Yes	1	No	0	No	0
The former Yugoslav Republic of	Yes	1	Yes	1	Yes	0	Yes	1	Yes	1	Yes	1	Yes	1	Yes	1
Turkey	Yes	1	Yes	1	Yes	0	Yes	1	No	0	Yes	1	Not applica	0	Not applicabl	0
Turkmenistan	Yes	1	Yes	1	No	1	Yes	1	Yes	1	Yes	1	Yes	1	Yes	1
Ukraine	Yes	1	No	0	Yes	0	No	0	No	0	No	0	No	0	No	0
United Kingdom	Yes	1	Yes	1	No	1	Yes	1	No	0	No	0	No	0	Yes	1
Uzbekistan	Yes	1	No	0	Yes	0	Yes	1	Yes	1	Yes	1	Not applica	0	No	0

	Taxation and price policies																	
	tax policies and, where appropriate, price policies on tobacco products so as to contribute to the health objectives aimed at reducing tobacco consumption?	score_taxpol_adv	prohibiting or restricting, as appropriate, sales to international travellers of tax- and duty-free tobacco products?	score_salesint_adv	prohibiting or restricting, as appropriate, imports by international travellers of tax- and duty-free tobacco products?	score_importsint_adv	requiring marking of all unit packets and packages of tobacco products and any outside packaging of such products to assist in determining the origin of the product?	score_marking_core	developing a practical tracking and tracing regime that would further secure the distribution system and assist in the investigation of illicit trade?	score_tracktrace_adv	requiring that marking is presented in legible form and/or appears in the principal language or languages of the country?	score_markleg_core	enacting or strengthening legislation, with appropriate penalties and remedies, against illicit trade in tobacco products, including counterfeit and contraband cigarettes?	score_legisltrade_core	prohibiting and/or promoting the prohibition of the distribution of free tobacco products: [to the public?]	score_freetob_core	prohibiting the sale of cigarettes individually or in small packets?	score_smallpack_adv
Albania	Yes	1	Yes	1	Yes	1	Yes	1	No	0	Yes	1	Yes	1	Yes	1	Yes	1
Andorra																		
Armenia	Yes	1	Yes	1	Yes	1	Yes	1	No	0	Yes	1	Yes	1	Yes	1	Yes	1
Austria	Yes	1	No	0	Yes	1	No	0	Yes	1	Yes	1	Yes	1	Yes	1	Yes	1
Azerbaijan	Yes	1	No	0	Yes	1	Yes	1	Yes	1	Yes	1	Yes	1	Yes	1	Yes	1
Belarus	Yes	1	Yes	1	Yes	1	Yes	1	Yes	1	Yes	1	Yes	1	Yes	1	Yes	1
Belgium	Yes	1	Yes	1	Yes	1	Yes	1	Yes	1	Yes	1	Yes	1	Yes	1	Yes	1
Bosnia and Herzegovina	Yes	1	No	0	No	0	Yes	1	No	0	Yes	1	Yes	1	Yes	1	Yes	1
Bulgaria	Yes	1	Yes	1	Yes	1	Yes	1	Yes	1	Yes	1	Yes	1	Yes	1	Yes	1
Croatia	Yes	1	No	0	No	0	Yes	1	No	0	Yes	1	Yes	1	Yes	1	Yes	1
Cyprus	No	0	No	0	No	0	Yes	1	Yes	1	Yes	1	Yes	1	Yes	1	Yes	1
Czech Republic	Yes	1	Yes	1	Yes	1	Yes	1	Yes	1	Yes	1	Yes	1	Yes	1	Yes	1
Denmark	Yes	1	No	0	Yes	1	Yes	1	Yes	1	Yes	1	Yes	1	Yes	1	Yes	1
Estonia	Yes	1	Yes	1	Yes	1	Yes	1	Yes	1	Yes	1	Yes	1	Yes	1	Yes	1
Finland	Yes	1	Yes	1	Yes	1	Yes	1	Yes	1	Yes	1	Yes	1	Yes	1	Yes	1
France	Yes	1	No	0	Yes	1	Yes	1	Yes	1	Yes	1	Yes	1	Yes	1	Yes	1
Georgia	Yes	1	Yes	1	Yes	1	Yes	1	Yes	1	Yes	1	Yes	1	Yes	1	Yes	1
Germany	Yes	1	No	0	Yes	1	No	0	No	0	Yes	1	Yes	1	Yes	1	Yes	1
Greece	Yes	1	Yes	1	Yes	1	No	0	No	0	Yes	1	Yes	1	Yes	1	Yes	1
Hungary	Yes	1	Yes	1	Yes	1	No	0	Yes	1	Yes	1	Yes	1	Yes	1	Yes	1
Iceland	Yes	1	Yes	1	Yes	1	No	0	No	0	N/A	0	No	0	Yes	1	Yes	1
Ireland	Yes	1	Yes	1	Yes	1	Yes	1	Yes	1	Yes	1	Yes	1	Yes	1	Yes	1
Israel	Yes	1	Yes	1	Yes	1	Yes	1	No	0	Yes	1	Yes	1	Yes	1	Yes	1
Italy	Yes	1	Yes	1	Yes	1	Yes	1	Yes	1	Yes	1	Yes	1	Yes	1	Yes	1
Kazakhstan	No	0	No	0	No	0	Yes	1	No	0	No	0	No	0	Yes	1	Yes	1
Kyrgyzstan	Yes	1	Yes	1	Yes	1	Yes	1	No	0	Yes	1	Yes	1	Yes	1	Yes	1
Latvia	Yes	1	No	0	No	0	Yes	1	Yes	1	Yes	1	Yes	1	Yes	1	Yes	1
Lithuania	Yes	1	No	0	Yes	1	Yes	1	Yes	1	Yes	1	Yes	1	Yes	1	Yes	1
Luxembourg	No	0	No	0	No	0	Yes	1	Yes	1	Yes	1	Yes	1	Yes	1	Yes	1
Malta	No	0	No	0	No	0	Yes	1	Yes	1	Yes	1	Yes	1	Yes	1	Yes	1
Monaco																		
Montenegro	No	0	No	0	No	0	Yes	1	Yes	1	No	0	Yes	1	Yes	1	Yes	1
Netherlands	Yes	1	No	0	No	0	Yes	1	Yes	1	Yes	1	Yes	1	Yes	1	Yes	1
Norway	Yes	1	Yes	1	Yes	1	Yes	1	No	0	Yes	1	Yes	1	Yes	1	Yes	1
Poland	No	0	No	0	No	0	Yes	1	Yes	1	Yes	1	Yes	1	No	0	No	0
Portugal	Yes	1	No	0	Yes	1	Yes	1	Yes	1	Yes	1	Yes	1	Yes	1	Yes	1
Republic of Moldova	Yes	1	Yes	1	Yes	1	Yes	1	No	0	Yes	1	Yes	1	Yes	1	Yes	1
Romania	Yes	1	Yes	1	Yes	1	Yes	1	No	0	Yes	1	Yes	1	No	0	Yes	1
Russian Federation	Yes	1	No	0	No	0	Yes	1	Yes	1	Yes	1	Yes	1	Yes	1	Yes	1
San Marino	No	0	No	0	No	0	No	0	No	0	No	0	No	0	Yes	1	Yes	1
Serbia	Yes	1	Yes	1	Yes	1	Yes	1	Yes	1	Yes	1	Yes	1	Yes	1	Yes	1
Slovakia	Yes	1	Yes	1	Yes	1	Yes	1	Yes	1	Yes	1	Yes	1	Yes	1	Yes	1
Slovenia	Yes	1	No	0	Yes	1	Yes	1	Yes	1	Yes	1	Yes	1	Yes	1	Yes	1
Spain	Yes	1	Yes	1	Yes	1	Yes	1	Yes	1	Yes	1	Yes	1	Yes	1	Yes	1
Sweden	Yes	1	Yes	1	Yes	1	Yes	1	Yes	1	Yes	1	Yes	1	Yes	1	Yes	1
Switzerland																		
Tajikistan	Yes	1	Yes	1	Yes	1	Yes	1	No	0	Yes	1	No	0	No	0	No	0
The former Yugoslav Republic of	Yes	1	Yes	1	Yes	1	Yes	1	N/A	0	Yes	1	Yes	1	Yes	1	Yes	1
Turkey	Yes	1	Yes	1	Yes	1	Yes	1	Yes	1	Yes	1	Yes	1	Yes	1	Yes	1
Turkmenistan	Yes	1	Yes	1	Yes	1	Yes	1	No	0	Yes	1	Yes	1	Yes	1	Yes	1
Ukraine	No	0	No	0	Yes	1	Yes	1	No	0	Yes	1	No	0	Yes	1	Yes	1
United Kingdom	Yes	1	No	0	Yes	1	Yes	1	Yes	1	Yes	1	Yes	1	Yes	1	Yes	1
Uzbekistan	Yes	1	No	0	No	0	No	0	No	0	Yes	1	Yes	1	Yes	1	Yes	1

	What is the type/nature of the measure providing for the ban? [national law]	score_natban_core	Please specify the settings and extent/comprehensiveness of measures applied in indoor workplaces, public transport, indoor public places and, as appropriate, other public places. Indoor workplaces: [government buildings]	score_govbuild_core	Please specify the settings and extent/comprehensiveness of measures applied in indoor workplaces, public transport, indoor public places and, as appropriate, other public places. Indoor workplaces: [health-care facilities]	score_healthfac_core	Please specify the settings and extent/comprehensiveness of measures applied in indoor workplaces, public transport, indoor public places and, as appropriate, other public places. Indoor workplaces: [educational facilities6]	score_educfac_core	Please specify the settings and extent/comprehensiveness of measures applied in indoor workplaces, public transport, indoor public places and, as appropriate, other public places. Indoor workplaces: [universities]	score_univ_core	Please specify the settings and extent/comprehensiveness of measures applied in indoor workplaces, public transport, indoor public places and, as appropriate, other public places. Indoor workplaces: [private workplaces]	score_privworkpl_core	Please specify the settings and extent/comprehensiveness of measures applied in indoor workplaces, public transport, indoor public places and, as appropriate, other public places. Public transport: [airplanes]	score_airplanes_core	Please specify the settings and extent/comprehensiveness of measures applied in indoor workplaces, public transport, indoor public places and, as appropriate, other public places. Public transport: [trains]	score_trains_core	specify the settings and extent/comprehensiveness of measures applied in indoor workplaces, public transport, indoor public places and, as appropriate, other public places. Public transport: [ground public transport (buses, trolleybuses, trams)]
Albania	Yes	1	Complete	1	Complete	1	Complete	1	Complete	1	Complete	1	Complete	1	Complete	1	Complete
Andorra																	
Armenia	Yes	1	Partial	0.5	Complete	1	Complete	1	Complete	1	Partial	0.5	Complete	1	Partial	0.5	Complete
Austria	Yes	1	Complete	1	Partial	0.5	Complete	1	Partial	0.5	Partial	0.5	Complete	1	Complete	1	Complete
Azerbaijan	Yes	1	Partial	0.5	Complete	1	Complete	1	Complete	1	Partial	0.5	Complete	1	Partial	0.5	Complete
Belarus	Yes	1	Partial	0.5	Complete	1	Complete	1	Complete	1	Complete	1	Complete	1	Complete	1	Complete
Belgium	Yes	1	Complete	1	Complete	1	Complete	1	Complete	1	Complete	1	Complete	1	Complete	1	Complete
Bosnia and Herzegovina	No	0	Partial	0.5	Partial	0.5	Partial	0.5	Partial	0.5	Partial	0.5	Complete	1	Complete	1	Complete
Bulgaria	Yes	1	Complete	1	Complete	1	Complete	1	Complete	1	Complete	1	Complete	1	Complete	1	Complete
Croatia	Yes	1	Complete	1	Complete	1	Complete	1	Complete	1	Complete	1	Complete	1	Complete	1	Complete
Cyprus	Yes	1	Complete	1	Complete	1	Complete	1	Complete	1	Complete	1	Complete	1	Complete	1	Complete
Czech Republic	Yes	1	Partial	0.5	Complete	1	Complete	1	Complete	1	Partial	0.5	Complete	1	Complete	1	Complete
Denmark	Yes	1	Partial	0.5	Partial	0.5	Complete	1	Partial	0.5	Partial	0.5	Complete	1	Complete	1	Complete
Estonia	Yes	1	Partial	0.5	Partial	0.5	Complete	1	Partial	0.5	None	0	Complete	1	Partial	0.5	Complete
Finland	Yes	1	Partial	0.5	Partial	0.5	Complete	1	Partial	0.5	Partial	0.5	Complete	1	Partial	0.5	Partial
France	Yes	1	Complete	1	Complete	1	Complete	1	Complete	1	Partial	0.5	Complete	1	Complete	1	Complete
Georgia	Yes	1	Complete	1	Complete	1	Complete	1	Complete	1	Complete	1	Complete	1	Complete	1	Complete
Germany	Yes	1	Complete	1	Complete	1	Partial	0.5	Partial	0.5	Partial	0.5	Complete	1	Complete	1	Complete
Greece	Yes	1	Complete	1	Complete	1	Complete	1	Complete	1	Complete	1	Complete	1	Complete	1	Complete
Hungary	Yes	1	Complete	1	Complete	1	Complete	1	Complete	1	Complete	1	Partial	0.5	Complete	1	Complete
Iceland	Yes	1	Complete	1	Complete	1	Complete	1	Complete	1	Partial	0.5	Complete	1	Complete	1	None
Ireland	Yes	1	Complete	1	Complete	1	Complete	1	Complete	1	Complete	1	Complete	1	Complete	1	Complete
Israel	Yes	1	Complete	1	Complete	1	Complete	1	Partial	0.5	Partial	0.5	Complete	1	Complete	1	Complete
Italy	Yes	1	Partial	0.5	Partial	0.5	Complete	1	Partial	0.5	Partial	0.5	Complete	1	Complete	1	Complete
Kazakhstan	Yes	1	Complete	1	Complete	1	Complete	1	Complete	1	Complete	1	Complete	1	Partial	0.5	Partial
Kyrgyzstan	Yes	1	Complete	1	Complete	1	Complete	1	Complete	1	Complete	1	Complete	1	Partial	0.5	Complete
Latvia	Yes	1	Complete	1	Partial	0.5	Complete	1	Complete	1	Partial	0.5	Complete	1	Partial	0.5	Complete
Lithuania	Yes	1	Partial	0.5	Complete	1	Complete	1	Complete	1	Partial	0.5	Partial	0.5	Partial	0.5	Complete
Luxembourg	Yes	1	Complete	1	Complete	1	Complete	1	Complete	1	Partial	0.5	Complete	1	Complete	1	Complete
Malta	Yes	1	Complete	1	Complete	1	Complete	1	Complete	1	Complete	1	Complete	1	None	0	Complete
Monaco																	
Montenegro	Yes	1	Complete	1	Complete	1	Complete	1	Complete	1	Complete	1	Complete	1	Complete	1	Complete
Netherlands	Yes	1	Complete	1	Complete	1	Complete	1	Complete	1	None	0	Complete	1	Complete	1	Complete
Norway	Yes	1	Complete	1	Complete	1	Complete	1	Complete	1	Complete	1	Complete	1	Complete	1	Complete
Poland		0		0		0		0		0		0	Complete	1	Complete	1	Complete
Portugal	Yes	1	Complete	1	Complete	1	Complete	1	Complete	1	Complete	1	Complete	1	Complete	1	Complete
Republic of Moldova	Yes	1	Complete	1	Complete	1	Complete	1	Complete	1	Complete	1	Complete	1	Complete	1	Complete
Romania	Yes	1	Partial	0.5	None	0	Partial	0.5	Partial	0.5	Partial	0.5	Complete	1	Complete	1	Complete
Russian Federation	Yes	1	Complete	1	Complete	1	Complete	1	Complete	1	Complete	1	Complete	1	Complete	1	Complete
San Marino	Yes	1	Complete	1	Complete	1	Complete	1	Complete	1	Complete	1	None	0	None	0	Complete
Serbia	Yes	1	Complete	1	Complete	1	Complete	1	Complete	1	Partial	0.5	Complete	1	Complete	1	Complete
Slovakia	Yes	1	Complete	1	Complete	1	Complete	1	Complete	1	Complete	1	Complete	1	Complete	1	Complete
Slovenia	Yes	1	Complete	1	Complete	1	Complete	1	Complete	1	Complete	1	Complete	1	Complete	1	Complete
Spain	Yes	1	Complete	1	Complete	1	Complete	1	Complete	1	Complete	1	Complete	1	Complete	1	Complete
Sweden	Yes	1	Partial	0.5	Partial	0.5	Partial	0.5	Partial	0.5	Partial	0.5	Complete	1	Complete	1	Complete
Switzerland																	
Tajikistan	Yes	1	Partial	0.5	Partial	0.5	Partial	0.5	Partial	0.5	Partial	0.5	Complete	1	Complete	1	Complete
The former Yugoslav Republic of	Yes	1	Complete	1	Complete	1	Complete	1	Complete	1	Complete	1	Complete	1	Complete	1	Complete
Turkey	Yes	1	Complete	1	Complete	1	Complete	1	Complete	1	Complete	1	Complete	1	Complete	1	Complete
Turkmenistan	Yes	1	Complete	1	Complete	1	Complete	1	Complete	1	Complete	1	Complete	1	Complete	1	Complete
Ukraine	Yes	1	Complete	1	Complete	1	Complete	1	Complete	1	Partial	0.5	Complete	1	Complete	1	Complete
United Kingdom	Yes	1	Complete	1	Complete	1	Complete	1	Complete	1	Complete	1	Complete	1	Complete	1	Complete
Uzbekistan	Yes	1	Complete	1	Partial	0.5	Complete	1	Complete	1	Partial	0.5	Complete	1	Partial	0.5	Partial

Note: highlighted are the indicators manually updated as per the information reported by the partners in WP9

Other national key regulations

score_gptransp_core	specify the settings and extent/comprehensiveness of measures applied in indoor workplaces, public transport, indoor public places and, as appropriate, other public places. Public transport: [motor vehicles used as places of work (taxis, ambulanc	score_motorwork_core	Please specify the settings and extent/comprehensiveness of measures applied in indoor workplaces, public transport, indoor public places and, as appropriate, other public places. Public transport: [private vehicles]	score_privateveh_core	Please specify the settings and extent/comprehensiveness of measures applied in indoor workplaces, public transport, indoor public places and, as appropriate, other public places. Indoor public places: [cultural facilities]	score_cultural_core	Please specify the settings and extent/comprehensiveness of measures applied in indoor workplaces, public transport, indoor public places and, as appropriate, other public places. Indoor public places: [shopping malls]	score_malls_core	Please specify the settings and extent/comprehensiveness of measures applied in indoor workplaces, public transport, indoor public places and, as appropriate, other public places. Indoor public places: [pubs and bars]	score_pubs_core	Please specify the settings and extent/comprehensiveness of measures applied in indoor workplaces, public transport, indoor public places and, as appropriate, other public places. Indoor public places: [nightclubs]	score_clubs_core	Please specify the settings and extent/comprehensiveness of measures applied in indoor workplaces, public transport, indoor public places and, as appropriate, other public places. Indoor public places: [restaurants]	score_restaurants_core
1	Complete	1	None	0	Complete	1	Complete	1	Complete	1	Complete	1	Complete	1
1	Complete	1	Complete	1	Complete	1	Partial	0.5	Partial	0.5	Partial	0.5	Partial	0.5
1	Complete	1	Partial	0.5	Partial	0.5	Partial	0.5	Complete	1	Complete	1	Complete	1
1	Complete	1	None	0	Complete	1	Partial	0.5	Partial	0.5	Partial	0.5	Partial	0.5
1	Partial	0.5	Partial	0.5	Complete	1	Complete	1	Partial	0.5	Partial	0.5	Partial	0.5
1	Complete	1	Complete	1	Complete	1	Complete	1	Complete	1	Complete	1	Complete	1
1	Complete	1	None	0	Complete	1	Partial	0.5	None	0	None	0	Partial	0.5
1	Complete	1	None	0	Complete	1	Complete	1	Complete	1	Complete	1	Complete	1
1	Complete	1	None	0	Complete	1	Complete	1	Partial	0.5	Partial	0.5	Complete	1
1	Complete	1	Partial	0.5	Complete	1	Complete	1	Complete	1	Complete	1	Complete	1
1	Complete	1	None	0	Complete	1	Partial	0.5	Partial	0.5	Partial	0.5	Partial	0.5
1	Partial	0.5	None	0	Partial	0.5	Partial	0.5	Partial	0.5	Partial	0.5	Partial	0.5
1	Complete	1	None	0	Partial	0.5	Partial	0.5	Partial	0.5	Partial	0.5	Partial	0.5
0.5	Partial	0.5	Partial	0.5	Partial	0.5	Partial	0.5	Partial	0.5	Partial	0.5	Partial	0.5
1	Complete	1	Partial	0.5	Partial	0.5	Partial	0.5	Partial	0.5	Partial	0.5	Partial	0.5
1	None	0	None	0	Complete	1	Complete	1	Complete	1	Complete	1	Complete	1
1	Complete	1	None	0	Partial	0.5	Partial	0.5	Partial	0.5	Partial	0.5	Partial	0.5
1	Complete	1	Complete	1	Complete	1	Complete	1	Complete	1	Complete	1	Complete	1
1	Partial	0.5	None	0	Complete	1	Complete	1	Complete	1	Complete	1	Complete	1
0	Complete	1	None	0	Complete	1	Complete	1	None	0	Complete	1	Complete	1
1	Complete	1	Partial	0.5	Complete	1	Complete	1	Complete	1	Complete	1	Complete	1
1	Complete	1	None	0	Partial	0.5	Partial	0.5	Partial	0.5	Partial	0.5	Partial	0.5
1	Complete	1	Complete	1	Partial	0.5	Partial	0.5	Partial	0.5	Partial	0.5	Partial	0.5
0.5	Partial	0.5	None	0	Complete	1	Complete	1	Partial	0.5	Partial	0.5	Partial	0.5
1	Complete	1	None	0	Complete	1	Complete	1	Complete	1	Complete	1	Partial	0.5
1	Complete	1	Partial	0.5	Complete	1	Complete	1	Complete	1	Complete	1	Complete	1
1	Complete	1	Partial	0.5	Complete	1	Complete	1	Complete	1	Complete	1	Complete	1
1	Complete	1	Partial	0.5	Complete	1	Complete	1	Complete	1	Complete	1	Complete	1
1	Complete	1	Partial	0.5	Complete	1	Complete	1	Complete	1	Complete	1	Complete	1
1	Complete	1	Complete	1	Complete	1	Complete	1	Complete	1	Complete	1	Complete	1
1	Complete	1	None	0	Complete	1	Complete	1	Complete	1	Complete	1	Complete	1
1	Complete	1	None	0	Complete	1	Complete	1	Complete	1	Complete	1	Complete	1
1	Complete	1	None	0	Complete	1	Complete	1	Partial	0.5	Partial	0.5	Partial	0.5
1	Complete	1	None	0	Complete	1	Partial	0.5	Partial	0.5	Partial	0.5	Partial	0.5
1	Complete	1	Partial	0.5	Complete	1	Complete	1	Complete	1	Complete	1	Complete	1
1	None	0	None	0	Partial	0.5	Partial	0.5	Partial	0.5	Partial	0.5	Partial	0.5
1	Complete	1	None	0	Complete	1	Complete	1	Complete	1	Complete	1	Complete	1
1	Complete	1	Complete	1	Complete	1	Complete	1	Partial	0.5	Partial	0.5	Partial	0.5
1	Complete	1	None	0	Complete	1	Complete	1	Partial	0.5	Partial	0.5	Partial	0.5
1	Complete	1	Partial	0.5	Complete	1	Complete	1	None	0	None	0	Partial	0.5
1	Complete	1	Partial	0.5	Complete	1	Complete	1	Complete	1	Complete	1	Complete	1
1	Complete	1	None	0	Complete	1	Complete	1	Complete	1	Complete	1	Complete	1
1	Partial	0.5	None	0	Partial	0.5	Partial	0.5	Partial	0.5	Partial	0.5	Partial	0.5
1	Partial	0.5	Partial	0.5	Partial	0.5	Partial	0.5	Partial	0.5	Partial	0.5	Partial	0.5
1	Complete	1		0	Complete	1	Complete	1	Complete	1	Complete	1	Complete	1
1	Complete	1	Partial	0.5	Complete	1	Complete	1	Complete	1	Complete	1	Complete	1
1	Complete	1	Complete	1	Complete	1	Complete	1	Complete	1	Complete	1	Complete	1
1	Partial	0.5	None	0	Complete	1	Partial	0.5	Complete	1	Complete	1	Complete	1
1	Complete	1	Partial	0.5	Complete	1	Complete	1	Complete	1	Complete	1	Complete	1
0.5	Partial	0.5	None	0	Partial	0.5	Complete	1	Partial	0.5	Partial	0.5	Partial	0.5

testing and measuring the contents of tobacco products?	score_testing_contents_core	testing and measuring the emissions of tobacco products?	score_testing_emissions_core	regulating the contents of tobacco products?	score_regulation_contents_core	regulating the emissions of tobacco products?	score_regulation_emmissions_core	requiring manufacturers or importers of tobacco products to disclose to Government authorities information about the: [contents of tobacco products?]	score_disclose_contents_core	requiring manufacturers or importers of tobacco products to disclose to Government authorities information about the: [emissions of tobacco products?]	score_disclose_emissions_core	requiring that packaging and labelling do not promote a product by any means that are false, misleading, deceptive or likely to create an erroneous impression about its characteristics, health effects, hazards or emissions?	score_misl_core	requiring that each unit packet and package of tobacco products and any outside packaging and labelling of such products carry health warnings describing the harmful effects of tobacco use?	score_healthwarning_core
Yes	1	Yes	1	Yes	1	No	0	Yes	1	Yes	1	Yes	1	Yes	1
Yes	1	Yes	1	Yes	1	Yes	1	No	0	Yes	1	Yes	1	Yes	1
Yes	1	Yes	1	Yes	1	Yes	1	Yes	1	Yes	1	Yes	1	Yes	1
Yes	1	No	0	Yes	1	No	0	Yes	1	Yes	1	Yes	1	Yes	1
Yes	1	Yes	1	Yes	1	Yes	1	Yes	1	Yes	1	Yes	1	Yes	1
Yes	1	Yes	1	Yes	1	Yes	1	Yes	1	Yes	1	Yes	1	Yes	1
Yes	1	Yes	1	Yes	1	No	0	Yes	1	Yes	1	Yes	1	Yes	1
Yes	1	Yes	1	Yes	1	Yes	1	Yes	1	Yes	1	Yes	1	Yes	1
Yes	1	Yes	1	Yes	1	Yes	1	Yes	1	Yes	1	Yes	1	Yes	1
No	0	No	0	Yes	1	Yes	1	Yes	1	Yes	1	Yes	1	Yes	1
Yes	1	Yes	1	Yes	1	Yes	1	Yes	1	Yes	1	Yes	1	Yes	1
Yes	1	Yes	1	Yes	1	Yes	1	Yes	1	Yes	1	Yes	1	Yes	1
Yes	1	Yes	1	Yes	1	Yes	1	Yes	1	Yes	1	Yes	1	Yes	1
Yes	1	Yes	1	Yes	1	Yes	1	Yes	1	Yes	1	Yes	1	Yes	1
Yes	1	Yes	1	Yes	1	Yes	1	Yes	1	Yes	1	Yes	1	Yes	1
Yes	1	Yes	1	Yes	1	Yes	1	Yes	1	Yes	1	Yes	1	Yes	1
Yes	1	Yes	1	Yes	1	Yes	1	Yes	1	Yes	1	Yes	1	Yes	1
No	0	Yes	1	Yes	1	Yes	1	Yes	1	Yes	1	Yes	1	Yes	1
Yes	1	Yes	1	Yes	1	Yes	1	Yes	1	Yes	1	Yes	1	Yes	1
Yes	1	Yes	1	Yes	1	No	0	Yes	1	Yes	1	Yes	1	Yes	1
Yes	1	Yes	1	Yes	1	Yes	1	Yes	1	Yes	1	Yes	1	Yes	1
Yes	1	Yes	1	No	0	No	0	Yes	1	Yes	1	Yes	1	Yes	1
Yes	1	Yes	1	Yes	1	Yes	1	Yes	1	Yes	1	Yes	1	Yes	1
Yes	1	Yes	1	Yes	1	Yes	1	Yes	1	Yes	1	Yes	1	Yes	1
No	0	No	0	Yes	1	Yes	1	Yes	1	Yes	1	Yes	1	Yes	1
No	0	Yes	1	Yes	1	Yes	1	Yes	1	Yes	1	Yes	1	Yes	1
Yes	1	No	0	Yes	1	No	0	Yes	1	Yes	1	Yes	1	Yes	1
No	0	No	0	Yes	1	Yes	1	Yes	1	Yes	1	Yes	1	Yes	1
No	0	No	0	Yes	1	Yes	1	Yes	1	Yes	1	Yes	1	Yes	1
Yes	1	Yes	1	Yes	1	Yes	1	Yes	1	Yes	1	Yes	1	Yes	1
Yes	1	Yes	1	Yes	1	Yes	1	Yes	1	Yes	1	Yes	1	Yes	1
No	0	No	0	No	0	Yes	1	Yes	1	Yes	1	Yes	1	Yes	1
Yes	1	Yes	1	Yes	1	Yes	1	Yes	1	Yes	1	No	0	No	0
Yes	1	Yes	1	Yes	1	Yes	1	Yes	1	Yes	1	Yes	1	Yes	1
Yes	1	Yes	1	Yes	1	Yes	1	Yes	1	Yes	1	Yes	1	Yes	1
No	0	Yes	1	Yes	1	Yes	1	Yes	1	Yes	1	Yes	1	Yes	1
Yes	1	Yes	1	Yes	1	Yes	1	Yes	1	Yes	1	Yes	1	Yes	1
No	0	No	0	No	0	No	0	No	0	No	0	No	0	No	0
No	0	Yes	1	No	0	Yes	1	No	0	No	0	Yes	1	Yes	1
Yes	1	Yes	1	Yes	1	Yes	1	No	0	No	0	Yes	1	Yes	1
Yes	1	Yes	1	Yes	1	Yes	1	Yes	1	Yes	1	Yes	1	Yes	1
Yes	1	Yes	1	Yes	1	Yes	1	Yes	1	Yes	1	Yes	1	Yes	1
Yes	1	Yes	1	Yes	1	Yes	1	Yes	1	Yes	1	Yes	1	Yes	1
No	0	No	0	No	0	No	0	No	0	No	0	Yes	1	Yes	1
Yes	1	Yes	1	Yes	1	Yes	1	Yes	1	Yes	1	Yes	1	Yes	1
Yes	1	Yes	1	Yes	1	No	0	Yes	1	Yes	1	Yes	1	Yes	1
Yes	1	Yes	1	Yes	1	Yes	1	Yes	1	Yes	1	Yes	1	Yes	1
No	0	Yes	1	No	0	Yes	1	Yes	1	Yes	1	Yes	1	Yes	1
Yes	1	Yes	1	Yes	1	Yes	1	Yes	1	Yes	1	Yes	1	Yes	1
No	0	No	0	Yes	1	No	0	Yes	1	No	0	No	0	Yes	1

ensuring that the health warnings are approved by the competent national authority?	score_healthwarningapp_core	ensuring that the health warnings are rotated?	score_healthwarningrot_core	ensuring that the health warnings are clear, visible and legible?	score_healthwarningvis_core	Does your law mandate, as a minimum, a style, size and colour of font to render the warning clear, visible and legible?	score_healthwarningmand_core	ensuring that the health warnings occupy 50% or more of the principal display areas?	score_healthwarningarea2_adv	requiring that the warnings and other textual information appear on each unit packet and package and on any outside packaging and labelling in the principal language or languages of the country?	score_lang_core	Does your ban cover: [display and visibility of tobacco products at points of sales?]	score_banpointssale_adv	Does your ban cover: [the domestic Internet?]	score_bandomint_core	Does your ban cover: [the global Internet?]	score_banglobint_core	Does your ban cover: [brand stretching and/or brand sharing?]	score_banglobint_adv
Yes	1	Yes	1	Yes	1	Yes	1	Yes	1	Yes	1	No	0	No	0	No	0	No	0
Yes	1	Yes	1	Yes	1	Yes	1	No	0	Yes	1	N/A	0	N/A	0	N/A	0	N/A	0
Yes	1	Yes	1	Yes	1	Yes	1	Yes	1	Yes	1	No	0	Yes	1	Yes	1	No	0
Yes	1	No	0	Yes	1	Yes	1	No	0	Yes	1	N/A	0	N/A	0	N/A	0	N/A	0
Yes	1	Yes	1	Yes	1	Yes	1	Yes	1	Yes	1	Yes	1	Yes	1	No	0	Yes	1
Yes	1	Yes	1	Yes	1	Yes	1	Yes	1	Yes	1	No	0	Yes	1	No	0	Yes	1
No	1	Yes	1	Yes	1	Yes	1	Yes	1	Yes	1	Yes	1	Yes	1	No	0	Yes	1
Yes	1	Yes	1	Yes	1	Yes	1	Yes	1	Yes	1	N/A	0	N/A	0	N/A	0	N/A	0
Yes	1	Yes	1	Yes	1	Yes	1	Yes	1	Yes	1	Yes	1	Yes	1	Yes	1	Yes	1
Yes	1	Yes	1	Yes	1	Yes	1	Yes	1	Yes	1	No	0	No	0	No	0	No	0
Yes	1	Yes	1	Yes	1	Yes	1	Yes	1	Yes	1	N/A	0	N/A	0	N/A	0	N/A	0
Yes	1	Yes	1	Yes	1	Yes	1	Yes	1	Yes	1	Yes	1	Yes	1	No	0	Yes	1
Yes	1	Yes	1	Yes	1	Yes	1	No	0	Yes	1	Yes	1	Yes	1	No	0	No	0
Yes	1	Yes	1	Yes	1	Yes	1	Yes	1	Yes	1	Yes	1	Yes	1	Yes	1	Yes	1
Yes	1	Yes	1	Yes	1	Yes	1	Yes	1	Yes	1	Yes	1	Yes	1	No	0	No	0
Yes	1	Yes	1	Yes	1	Yes	1	Yes	1	Yes	1	Yes	1	Yes	1	Yes	1	Yes	1
Yes	1	Yes	1	Yes	1	Yes	1	Yes	1	Yes	1	N/A	0	N/A	0	N/A	0	N/A	0
Yes	1	Yes	1	Yes	1	Yes	1	Yes	1	Yes	1	N/A	0	N/A	0	N/A	0	N/A	0
Yes	1	Yes	1	Yes	1	Yes	1	Yes	1	Yes	1	No	0	Yes	1	Yes	1	No	0
Yes	1	Yes	1	Yes	1	Yes	1	No	0	Yes	1	Yes	1	Yes	1	No	0	Yes	1
Yes	1	Yes	1	Yes	1	Yes	1	Yes	1	Yes	1	Yes	1	Yes	1	No	0	No	0
Yes	1	Yes	1	Yes	1	Yes	1	Yes	1	Yes	1	Yes	1	Yes	1	No	0	Yes	1
Yes	1	Yes	1	Yes	1	Yes	1	Yes	1	Yes	1	No	0	Yes	1	No	0	Yes	1
Yes	1	Yes	1	Yes	1	Yes	1	Yes	1	Yes	1	N/A	0	N/A	0	N/A	0	N/A	0
Yes	1	Yes	1	Yes	1	Yes	1	Yes	1	Yes	1	No	0	Yes	1	No	0	Yes	1
Yes	1	Yes	1	Yes	1	Yes	1	Yes	1	Yes	1	Yes	1	Yes	1	Yes	1	No	0
Yes	1	Yes	1	Yes	1	Yes	1	Yes	1	Yes	1	No	0	Yes	1	No	0	Yes	1
Yes	1	Yes	1	Yes	1	Yes	1	Yes	1	Yes	1	Yes	1	Yes	1	Yes	1	No	0
Yes	1	Yes	1	Yes	1	Yes	1	Yes	1	Yes	1	Yes	1	Yes	1	Yes	1	Yes	1
Yes	1	Yes	1	Yes	1	Yes	1	No	0	Yes	1	No	0	No	0	No	0	Yes	1
Yes	1	Yes	1	Yes	1	Yes	1	Yes	1	Yes	1	No	0	Yes	1	No	0	Yes	1
Yes	1	Yes	1	Yes	1	Yes	1	No	0	Yes	1	Yes	1	Yes	1	Yes	1	Yes	1
No	0	No	0	No	0	N/A	0	No	0	No	0	N/A	0	N/A	0	N/A	0	N/A	0
Yes	1	Yes	1	Yes	1	Yes	1	Yes	1	Yes	1	No	0	Yes	1	No	0	Yes	1
Yes	1	Yes	1	Yes	1	Yes	1	Yes	1	Yes	1	No	0	Yes	1	No	0	No	0
Yes	1	Yes	1	No	0	N/A	0	No	0	Yes	1	No	0	No	0	No	0	Yes	1
Yes	1	Yes	1	Yes	1	Yes	1	Yes	1	Yes	1	Yes	1	Yes	1	Yes	1	Yes	1
No	0	No	0	No	0	N/A	0	No	0	No	0	Yes	1	No	0	No	0	No	0
Yes	1	Yes	1	Yes	1	Yes	1	No	0	Yes	1	No	0	Yes	1	No	0	Yes	1
Yes	1	Yes	1	Yes	1	Yes	1	Yes	1	Yes	1	No	0	Yes	1	No	0	No	0
Yes	1	Yes	1	Yes	1	Yes	1	Yes	1	Yes	1	Yes	1	Yes	1	Yes	1	Yes	1
Yes	1	Yes	1	Yes	1	Yes	1	Yes	1	Yes	1	No	0	Yes	1	Yes	1	Yes	1
No	1	Yes	1	Yes	1	Yes	1	Yes	1	Yes	1	No	0	Yes	1	No	0	Yes	1
Yes	1	Yes	1	Yes	1	Yes	1	Yes	1	Yes	1	Yes	1	No	0	No	0	No	0
Yes	1	Yes	1	Yes	1	Yes	1	No	0	Yes	1	Yes	1	Yes	1	No	0	Yes	1
Yes	1	Yes	1	Yes	1	Yes	1	Yes	1	Yes	1	Yes	1	Yes	1	No	0	Yes	1
Yes	1	Yes	1	Yes	1	Yes	1	Yes	1	Yes	1	Yes	1	Yes	1	No	0	Yes	1
No	1	Yes	1	Yes	1	Yes	1	Yes	1	Yes	1	N/A	0	N/A	0	N/A	0	N/A	0
Yes	1	Yes	1	Yes	1	Yes	1	Yes	1	Yes	1	Yes	1	Yes	1	Yes	1	Yes	1
Yes	1	Yes	1	Yes	1	Yes	1	No	0	Yes	1	Yes	1	No	0	No	0	No	0

Does your ban cover: [product placement as a means of advertising or promotion?]	score_banprodplac_core	Does your ban cover: [the depiction of tobacco or tobacco use in entertainment media products?]	score_banentmedia_adv	Does your ban cover: [tobacco sponsorship of international events or activities and/or participants therein?]	score_banintevent_core	Does your ban cover: [contributions from tobacco companies to any other entity for "socially responsible causes" and/or any other activities implemented under the umbrella of "corporate social responsibility" by the tobacco industry?]	score_bancsr_adv	Does your ban cover: [cross-border advertising, promotion and sponsorship originating from your territory?]	score_bancrossborderwithin_core	Does your ban cover: [the same forms of cross-border advertising, promotion and sponsorship entering your territory for which domestic regulation apply?]	score_bancrossborderent_core	imposing penalties for cross-border advertising equal to those applicable to domestic advertising, promotion and sponsorship originating from your territory in accordance with national law?	score_banpenalties_core	prohibiting the sales of tobacco products to minors?	score_banminors_core	requiring that, in case of doubt, each seller of tobacco products requests that the purchaser provides appropriate evidence of having reached full legal age?	score_doubt_core	banning the sale of tobacco products in any manner by which they are directly accessible, such as open store shelves?	score_bandirectman_core
No	0	Yes	1	Yes	1	Yes	1	Yes	1	Yes	1	No	0	Yes	1	Yes	1	Yes	1
N/A	0	N/A	0	N/A	0	N/A	0	N/A	0	N/A	0	No	0	Yes	1	Yes	1	Yes	1
Yes	1	Yes	1	Yes	1	Yes	1	Yes	1	Yes	1	Yes	1	Yes	1	Yes	1	Yes	1
N/A	0	N/A	0	N/A	0	N/A	0	N/A	0	N/A	0	No	0	Yes	1	Yes	1	No	0
Yes	1	Yes	1	No	0	No	0	No	0	Yes	1	Yes	1	Yes	1	Yes	1	Yes	1
Yes	1	No	0	No	0	Yes	1	Yes	1	Yes	1	Yes	1	Yes	1	Yes	1	No	0
Yes	1	Yes	1	Yes	1	Yes	1	Yes	1	Yes	1	No	0	Yes	1	Yes	1	Yes	1
N/A	0	N/A	0	N/A	0	N/A	0	N/A	0	N/A	0	No	0	Yes	1	Yes	1	Yes	1
Yes	1	Yes	1	Yes	1	Yes	1	Yes	1	Yes	1	No	0	Yes	1	Yes	1	Yes	1
Yes	1	No	0	Yes	1	Yes	1	Yes	1	Yes	1	Yes	1	Yes	1	No	0	No	0
N/A	0	N/A	0	N/A	0	N/A	0	N/A	0	N/A	0	Yes	1	Yes	1	Yes	1	No	0
Yes	1	No	0	Yes	1	No	0	No	0	Yes	1	No	0	Yes	1	Yes	1	Yes	1
No	0	No	0	No	0	No	0	No	0	No	0	No	0	Yes	1	Yes	1	Yes	1
Yes	1	No	0	Yes	1	Yes	1	Yes	1	Yes	1	Yes	1	Yes	1	Yes	1	Yes	1
Yes	1	No	0	Yes	1	Yes	1	No	0	Yes	1	Yes	1	Yes	1	Yes	1	Yes	1
Yes	1	Yes	1	Yes	1	Yes	1	Yes	1	Yes	1	No	0	Yes	1	Yes	1	No	0
N/A	0	N/A	0	N/A	0	N/A	0	N/A	0	N/A	0	Yes	1	Yes	1	Yes	1	Yes	1
N/A	0	N/A	0	N/A	0	N/A	0	N/A	0	N/A	0	No	0	Yes	1	Yes	1	Yes	1
Yes	1	Yes	1	Yes	1	No	0	Yes	1	Yes	1	Yes	1	Yes	1	Yes	1	Yes	1
Yes	1	Yes	1	Yes	1	Yes	1	No	0	No	0	No	0	Yes	1	Yes	1	Yes	1
Yes	1	No	0	Yes	1	Yes	1	Yes	1	Yes	1	No	0	Yes	1	Yes	1	Yes	1
Yes	1	No	0	Yes	1	No	0	Yes	1	Yes	1	Yes	1	Yes	1	Yes	1	No	0
Yes	1	No	0	Yes	1	No	0	Yes	1	Yes	1	Yes	1	Yes	1	Yes	1	Yes	1
N/A	0	N/A	0	N/A	0	N/A	0	N/A	0	N/A	0	No	0	Yes	1	Yes	1	Yes	1
Yes	1	Yes	1	Yes	1	Yes	1	Yes	1	No	0	No	0	Yes	1	Yes	1	No	0
Yes	1	Yes	1	Yes	1	Yes	1	Yes	1	Yes	1	No	0	Yes	1	Yes	1	Yes	1
Yes	1	Yes	1	Yes	1	Yes	1	Yes	1	Yes	1	No	0	Yes	1	Yes	1	No	0
Yes	1	Yes	1	Yes	1	No	0	Yes	1	Yes	1	Yes	1	Yes	1	Yes	1	Yes	1
Yes	1	Yes	1	Yes	1	Yes	1	Yes	1	Yes	1	Yes	1	Yes	1	Yes	1	Yes	1
Yes	1	Yes	1	Yes	1	Yes	1	Yes	1	Yes	1	No	0	Yes	1	Yes	1	Yes	1
Yes	1	Yes	1	Yes	1	Yes	1	No	0	No	0	No	0	Yes	1	Yes	1	Yes	1
Yes	1	No	0	Yes	1	Yes	1	Yes	1	Yes	1	No	0	Yes	1	Yes	1	Yes	1
N/A	0	N/A	0	N/A	0	N/A	0	N/A	0	N/A	0	No	0	No	0	No	0	No	0
Yes	1	Yes	1	Yes	1	No	0	Yes	1	Yes	1	Yes	1	Yes	1	Yes	1	Yes	1
Yes	1	Yes	1	Yes	1	Yes	1	Yes	1	Yes	1	Yes	1	Yes	1	Yes	1	Yes	1
Yes	1	No	0	Yes	1	No	0	Yes	1	Yes	1	Yes	1	Yes	1	Yes	1	No	0
Yes	1	Yes	1	Yes	1	Yes	1	No	0	Yes	1	No	0	Yes	1	Yes	1	Yes	1
Yes	1	No	0	No	0	No	0	No	0	No	0	No	0	Yes	1	Yes	1	Yes	1
Yes	1	No	0	Yes	1	No	0	Yes	1	No	0	No	0	Yes	1	Yes	1	Yes	1
Yes	1	Yes	1	No	0	Yes	1	Yes	1	No	0	Yes	1	Yes	1	Yes	1	Yes	1
Yes	1	Yes	1	Yes	1	Yes	1	Yes	1	Yes	1	Yes	1	Yes	1	Yes	1	Yes	1
Yes	1	Yes	1	Yes	1	No	0	Yes	1	Yes	1	Yes	1	Yes	1	Yes	1	No	0
Yes	1	No	0	Yes	1	Yes	1	Yes	1	Yes	1	No	0	Yes	1	Yes	1	Yes	1
No	0	Yes	1	No	0	No	0	No	0	No	0	No	0	Yes	1	Yes	1	Yes	1
Yes	1	Yes	1	Yes	1	Yes	1	No	0	No	0	No	0	Yes	1	Yes	1	Yes	1
Yes	1	Yes	1	Yes	1	Yes	1	No	0	No	0	Yes	1	Yes	1	Yes	1	Yes	1
Yes	1	Yes	1	Yes	1	Yes	1	Yes	1	Yes	1	No	0	Yes	1	Yes	1	Yes	1
N/A	0	N/A	0	N/A	0	N/A	0	N/A	0	N/A	0	No	0	Yes	1	Yes	1	No	0
Yes	1	No	0	Yes	1	No	0	Yes	1	Yes	1	Yes	1	Yes	1	Yes	1	Yes	1
No	0	No	0	Yes	1	Yes	1	No	0	No	0	No	0	Yes	1	Yes	1	No	0

prohibiting the manufacture and sale of sweets, snacks, toys or any other objects in the form of tobacco products which appeal to minors?	score_toys_core	prohibiting the sale of tobacco products from vending machines?	score_toys_adv	providing for penalties against sellers and distributors in order to ensure compliance?	score_penaltsell_adv	**Public awareness raising and communications** requiring public disclosure of information about the: [contents of tobacco products?]	score_public_contents_core	requiring public disclosure of information about the: [emissions of tobacco products?]	score_public_emissions_core	education and public awareness programmes? (Please refer to programmes implemented since submission of your two-year report.)	score_educ_core	Do these education and public awareness programmes cover: [health risks of tobacco consumption?]	score_healthriskscons_core	Are the elaboration, management and implementation of communication, education, training and public awareness programmes guided by research and do they undergo pre-testing, monitoring and evaluation?	score_evaluation_adv	Are appropriate and special training or sensitization and awareness programmes on tobacco control addressed to: [health workers?]	score_healthw_core	Are appropriate and special training or sensitization and awareness programmes on tobacco control addressed to: [community workers?]	score_commw_core	Are appropriate and special training or sensitization and awareness programmes on tobacco control addressed to: [social workers?]	score_socw_core	Are appropriate and special training or sensitization and awareness programmes on tobacco control addressed to: [media professionals?]	score_medprof_core	Are appropriate and special training or sensitization and awareness programmes on tobacco control addressed to: [educators?]	score_educ_core	Are appropriate and special training or sensitization and awareness programmes on tobacco control addressed to: [decision-makers?]	score_decmak_core	Are appropriate and special training or sensitization and awareness programmes on tobacco control addressed to: [administrators?]	score_admin_core
No	0	Yes	1	Yes	1	Yes	1	No	0	Yes	1	Yes	1	Yes	1	Yes	1	Yes	1	Yes	1	Yes	1	Yes	1	Yes	1	Yes	1
Yes	1	Yes	1	Yes	1	No	0	Yes	1	Yes	1	Yes	1	Yes	1	Yes	1	Yes	1	Yes	1	Yes	1	Yes	1	Yes	1	Yes	1
No	0	No	0	Yes	1	Yes	1	Yes	1	Yes	1	Yes	1	Yes	1	Yes	1	Yes	1	Yes	1	Yes	1	Yes	1	Yes	1	No	0
No	0	Yes	1	Yes	1	Yes	1	Yes	1	Yes	1	Yes	1	No	0	Yes	1	Yes	1	No	0	Yes	1	Yes	1	Yes	1	No	0
Yes	1	Yes	1	Yes	1	Yes	1	Yes	1	Yes	1	Yes	1	Yes	1	Yes	1	No	0	Yes	1	Yes	1	Yes	1	Yes	1	Yes	1
No	0	No	0	Yes	1	Yes	1	Yes	1	Yes	1	Yes	1	Yes	1	Yes	1	No	0	No	0	No	0	Yes	1	No	0	No	0
Yes	1	Yes	1	Yes	1	Yes	1	Yes	1	Yes	1	Yes	1	No	0	Yes	1	Yes	1	No	0	No	0	Yes	1	Yes	1	Yes	1
No	0	Yes	1	Yes	1	Yes	1	Yes	1	Yes	1	Yes	1	Yes	1	Yes	1	Yes	1	Yes	1	Yes	1	Yes	1	Yes	1	Yes	1
Yes	1	Yes	1	Yes	1	Yes	1	Yes	1	Yes	1	Yes	1	Yes	1	Yes	1	No	0	No	0	Yes	1	Yes	1	Yes	1	Yes	1
Yes	1	Yes	1	Yes	1	Yes	1	Yes	1	Yes	1	Yes	1	Yes	1	Yes	1	Yes	1	Yes	1	No	0	Yes	1	No	0	No	0
Yes	1	No	0	Yes	1	Yes	1	Yes	1	Yes	1	Yes	1	No	0	Yes	1	No	0	No	0	No	0	Yes	1	No	0	No	0
No	0	No	0	Yes	1	Yes	1	Yes	1	No	0	N/A	0	N/A	0	No	0	No	0	No	0	No	0	No	0	No	0	No	0
Yes	1	Yes	1	Yes	1	Yes	1	Yes	1	Yes	1	Yes	1	Yes	1	Yes	1	Yes	1	Yes	1	No	0	No	0	No	0	No	0
No	0	Yes	1	Yes	1	Yes	1	Yes	1	Yes	1	Yes	1	Yes	1	Yes	1	No	0	No	0	No	0	Yes	1	No	0	No	0
No	0	Yes	1	Yes	1	Yes	1	Yes	1	Yes	1	Yes	1	Yes	1	No	0	No	0	No	0	No	0	No	0	No	0	No	0
Yes	1	Yes	1	Yes	1	No	0	No	0	Yes	1	Yes	1	Yes	1	Yes	1	No	0	Yes	1	Yes	1	Yes	1	Yes	1	Yes	1
No	0	No	0	Yes	1	Yes	1	Yes	1	Yes	1	Yes	1	Yes	1	Yes	1	Yes	1	Yes	1	No	0	Yes	1	Yes	1	No	0
Yes	1	Yes	1	Yes	1	Yes	1	Yes	1	Yes	1	Yes	1	Yes	1	Yes	1	Yes	1	Yes	1	No	0	Yes	1	No	0	Yes	1
No	0	Yes	1	Yes	1	Yes	1	Yes	1	Yes	1	Yes	1	Yes	1	Yes	1	No	0	No	0	No	0	Yes	1	No	0	No	0
Yes	1	Yes	1	Yes	1	No	0	No	0	Yes	1	Yes	1	No	0	No	0	No	0	No	0	No	0	Yes	1	No	0	No	0
Yes	1	No	0	Yes	1	Yes	1	Yes	1	Yes	1	Yes	1	Yes	1	Yes	1	Yes	1	Yes	1	Yes	1	Yes	1	Yes	1	Yes	1
Yes	1	Yes	1	Yes	1	Yes	1	Yes	1	Yes	1	Yes	1	Yes	1	Yes	1	No	0	No	0	No	0	Yes	1	No	0	No	0
No	0	No	0	Yes	1	Yes	1	Yes	1	Yes	1	Yes	1	Yes	1	Yes	1	No	0	No	0	No	0	No	0	No	0	No	0
Yes	1	Yes	1	Yes	1	No	0	No	0	Yes	1	Yes	1	Yes	1	Yes	1	No	0	Yes	1	Yes	1	No	0	Yes	1	No	0
Yes	1	Yes	1	Yes	1	Yes	1	Yes	1	Yes	1	Yes	1	Yes	1	Yes	1	Yes	1	Yes	1	Yes	1	Yes	1	Yes	1	Yes	1
Yes	1	Yes	1	Yes	1	Yes	1	Yes	1	Yes	1	Yes	1	Yes	1	Yes	1	No	0	No	0	No	0	Yes	1	No	0	No	0
Yes	1	Yes	1	Yes	1	Yes	1	Yes	1	Yes	1	Yes	1	No	0	No	0	No	0	No	0	No	0	Yes	1	No	0	No	0
Yes	1	No	0	Yes	1	No	0	No	0	Yes	1	Yes	1	Yes	1	Yes	1	No	0	No	0	No	0	No	0	No	0	No	0
Yes	1	No	0	Yes	1	Yes	1	Yes	1	Yes	1	Yes	1	Yes	1	Yes	1	Yes	1	No	0	No	0	Yes	1	Yes	1	Yes	1
Yes	1	Yes	1	Yes	1	No	0	No	0	Yes	1	Yes	1	Yes	1	Yes	1	No	0	No	0	No	0	Yes	1	Yes	1	No	0
No	0	No	0	Yes	1	Yes	1	Yes	1	Yes	1	Yes	1	Yes	1	Yes	1	Yes	1	Yes	1	No	0	Yes	1	No	0	No	0
Yes	1	Yes	1	Yes	1	Yes	1	Yes	1	Yes	1	Yes	1	Yes	1	Yes	1	Yes	1	No	0	No	0	Yes	1	No	0	No	0
No	0	No	0	No	0	Yes	1	Yes	1	Yes	1	Yes	1	Yes	1	Yes	1	No	0	No	0	No	0	Yes	1	No	0	No	0
Yes	1	No	0	Yes	1	Yes	1	Yes	1	Yes	1	Yes	1	Yes	1	Yes	1	Yes	1	Yes	1	No	0	Yes	1	No	0	No	0
Yes	1	Yes	1	Yes	1	Yes	1	Yes	1	Yes	1	Yes	1	Yes	1	Yes	1	No	0	No	0	Yes	1	Yes	1	No	0	No	0
Yes	1	Yes	1	Yes	1	Yes	1	Yes	1	Yes	1	Yes	1	Yes	1	No	0	No	0	No	0	No	0	No	0	No	0	No	0
Yes	1	Yes	1	Yes	1	Yes	1	No	0	Yes	1	Yes	1	Yes	1	No	0	No	0	No	0	No	0	No	0	No	0	No	0
No	0	No	0	Yes	1	No	0	No	0	Yes	1	Yes	1	Yes	1	Yes	1	No	0	Yes	1	No	0	Yes	1	Yes	1	Yes	1
Yes	1	Yes	1	Yes	1	No	0	No	0	Yes	1	Yes	1	Yes	1	Yes	1	No	0	No	0	No	0	Yes	1	No	0	No	0
Yes	1	Yes	1	Yes	1	No	0	No	0	Yes	1	Yes	1	No	0	Yes	1	No	0	No	0	Yes	1	No	0	Yes	1	No	0
Yes	1	Yes	1	Yes	1	Yes	1	Yes	1	Yes	1	Yes	1	Yes	1	Yes	1	Yes	1	Yes	1	Yes	1	Yes	1	No	0	No	0
No	0	No	0	Yes	1	Yes	1	Yes	1	Yes	1	Yes	1	Yes	1	Yes	1	Yes	1	Yes	1	Yes	1	Yes	1	Yes	1	Yes	1
No	0	No	0	Yes	1	Yes	1	No	0	Yes	1	No	0	Yes	1	Yes	1	Yes	1	Yes	1	Yes	1	Yes	1	Yes	1	Yes	1
No	0	No	0	No	0	No	0	No	0	Yes	1	Yes	1	Yes	1	Yes	1	Yes	1	Yes	1	Yes	1	Yes	1	Yes	1	Yes	1
Yes	1	Yes	1	Yes	1	Yes	1	Yes	1	Yes	1	Yes	1	Yes	1	Yes	1	Yes	1	Yes	1	Yes	1	Yes	1	Yes	1	Yes	1
Yes	1	Yes	1	Yes	1	Yes	1	Yes	1	Yes	1	Yes	1	Yes	1	Yes	1	Yes	1	Yes	1	Yes	1	Yes	1	Yes	1	Yes	1
Yes	1	Yes	1	Yes	1	No	0	Yes	1	Yes	1	Yes	1	Yes	1	Yes	1	No	0	Yes	1	Yes	1	Yes	1	Yes	1	Yes	1
Yes	1	Yes	1	Yes	1	Yes	1	Yes	1	Yes	1	Yes	1	No	0	No	0	No	0	No	0	No	0	No	0	No	0	No	0
No	0	Yes	1	Yes	1	No	0	No	0	Yes	1	Yes	1	Yes	1	Yes	1	Yes	1	No	0	No	0	Yes	1	No	0	Yes	1
Yes	1	Yes	1	Yes	1	Yes	1	No	0	Yes	1	Yes	1	Yes	1	Yes	1	Yes	1	Yes	1	Yes	1	Yes	1	Yes	1	No	0

**Monitoring** requiring the monitoring and collection of data on cross-border trade in tobacco products, including illicit trade?	score_monittrade_core	developing and/or promoting research that addresses: [determinants of tobacco consumption?]	score_determinantscons_core	developing and/or promoting research that addresses: [consequences of tobacco consumption?]	score_conseqcons_core	developing and/or promoting research that addresses: [the determinants and consequences of exposure to tobacco smoke?]	score_detconseqcons_core	a national system for epidemiological surveillance of: [patterns of tobacco consumption?]	score_patterns_core	a national system for epidemiological surveillance of: [determinants of tobacco consumption?]	score_natsysdet_core	a national system for epidemiological surveillance of: [consequences of tobacco consumption?]	score_natsyscons_core	a national system for epidemiological surveillance of: [exposure to tobacco smoke?]	score_natsysexp_core
No	0	Yes	1	Yes	1	Yes	1	Yes	1	Yes	1	Yes	1	Yes	1
No	0	Yes	1	Yes	1	Yes	1	Yes	1	Yes	1	Yes	1	Yes	1
Yes	1	Yes	1	Yes	1	No	0	Yes	1	Yes	1	Yes	1	Yes	1
Yes	1	Yes	1	No	0	Yes	1	No	0	Yes	1	No	0	No	0
Yes	1	Yes	1	Yes	1	Yes	1	Yes	1	No	0	Yes	1	Yes	1
Yes	1	Yes	1	Yes	1	No	0	Yes	1	Yes	1	Yes	1	Yes	1
Yes	1	Yes	1	Yes	1	Yes	1	Yes	1	Yes	1	Yes	1	Yes	1
Yes	1	Yes	1	Yes	1	Yes	1	Yes	1	Yes	1	No	0	Yes	1
Yes	1	Yes	1	Yes	1	Yes	1	Yes	1	Yes	1	Yes	1	Yes	1
Yes	1	No	0	No	0	No	0	Yes	1	No	0	No	0	No	0
No	0	Yes	1	Yes	1	Yes	1	Yes	1	Yes	1	No	0	Yes	1
No	0	No	0	No	0	No	0	Yes	1	No	0	Yes	1	No	0
Yes	1	No	0	No	0	No	0	Yes	1	No	0	Yes	1	Yes	1
Yes	1	Yes	1	Yes	1	Yes	1	Yes	1	Yes	1	Yes	1	Yes	1
Yes	1	Yes	1	Yes	1	Yes	1	Yes	1	Yes	1	Yes	1	Yes	1
No	0	Yes	1	No	0	No	0	Yes	1	Yes	1	No	0	Yes	1
Yes	1	Yes	1	Yes	1	Yes	1	Yes	1	Yes	1	Yes	1	Yes	1
No	0	Yes	1	Yes	1	Yes	1	Yes	1	No	0	No	0	Yes	1
Yes	1	Yes	1	Yes	1	Yes	1	Yes	1	Yes	1	No	0	Yes	1
No	0	Yes	1	Yes	1	No	0	Yes	1	Yes	1	No	0	No	0
Yes	1	Yes	1	Yes	1	Yes	1	Yes	1	Yes	1	Yes	1	Yes	1
Yes	1	Yes	1	Yes	1	Yes	1	Yes	1	Yes	1	Yes	1	Yes	1
Yes	1	Yes	1	Yes	1	Yes	1	Yes	1	Yes	1	Yes	1	Yes	1
No	0	Yes	1	Yes	1	Yes	1	Yes	1	Yes	1	Yes	1	Yes	1
Yes	1	Yes	1	Yes	1	Yes	1	Yes	1	Yes	1	No	0	No	0
Yes	1	Yes	1	Yes	1	Yes	1	Yes	1	Yes	1	Yes	1	Yes	1
Yes	1	Yes	1	Yes	1	No	0	Yes	1	No	0	Yes	1	No	0
Yes	1	No	0	No	0	No	0	Yes	1	No	0	Yes	1	No	0
Yes	1	Yes	1	Yes	1	Yes	1	Yes	1	Yes	1	Yes	1	Yes	1
Yes	1	Yes	1	Yes	1	Yes	1	Yes	1	Yes	1	No	0	Yes	1
Yes	1	Yes	1	Yes	1	Yes	1	Yes	1	Yes	1	Yes	1	Yes	1
Yes	1	Yes	1	Yes	1	Yes	1	Yes	1	Yes	1	Yes	1	Yes	1
Yes	1	Yes	1	No	0	Yes	1	Yes	1	Yes	1	Yes	1	Yes	1
Yes	1	Yes	1	Yes	1	Yes	1	Yes	1	Yes	1	Yes	1	Yes	1
Yes	1	Yes	1	Yes	1	Yes	1	Yes	1	Yes	1	Yes	1	Yes	1
No	0	No	0	No	0	No	0	Yes	1	No	0	No	0	Yes	1
No	0	Yes	1	Yes	1	No	0	Yes	1	Yes	1	Yes	1	Yes	1
No	0	No	0	No	0	No	0	No	0	No	0	No	0	No	0
Yes	1	Yes	1	Yes	1	Yes	1	Yes	1	Yes	1	Yes	1	Yes	1
Yes	1	Yes	1	Yes	1	No	0	Yes	1	No	0	Yes	1	Yes	1
Yes	1	Yes	1	Yes	1	Yes	1	Yes	1	Yes	1	Yes	1	Yes	1
Yes	1	Yes	1	Yes	1	Yes	1	Yes	1	Yes	1	Yes	1	Yes	1
Yes	1	Yes	1	Yes	1	Yes	1	Yes	1	Yes	1	Yes	1	Yes	1
Yes	1	No	0	No	0	No	0	No	0	No	0	No	0	No	0
Yes	1	Yes	1	Yes	1	Yes	1	Yes	1	Yes	1	Yes	1	Yes	1
Yes	1	Yes	1	Yes	1	Yes	1	Yes	1	Yes	1	Yes	1	Yes	1
No	0	Yes	1	Yes	1	Yes	1	Yes	1	Yes	1	Yes	1	Yes	1
Yes	1	No	0	Yes	1	Yes	1	Yes	1	No	0	No	0	No	0
Yes	1	Yes	1	Yes	1	Yes	1	Yes	1	Yes	1	Yes	1	Yes	1
No	0	Yes	1	No	0	No	0	Yes	1	Yes	1	No	0	Yes	1

**Cessation** developing and disseminating appropriate, comprehensive and integrated guidelines based on scientific evidence and best practices?	programmes to promote cessation of tobacco use, including: [media campaigns emphasizing the importance of quitting?]	programmes to promote cessation of tobacco use, including: [telephone quitlines?]	programmes to promote cessation of tobacco use, including: [local events, such as activities related to World No Tobacco Day or National No Smoking Day, if appropriate?]	programmes to promote cessation of tobacco use, including: [other (please specify)?]	design and implementation of programmes aimed at promoting the cessation of tobacco use, in such locations as: [educational institutions?]	design and implementation of programmes aimed at promoting the cessation of tobacco use, in such locations as: [health-care facilities?]	design and implementation of programmes aimed at promoting the cessation of tobacco use, in such locations as: [workplaces?]	design and implementation of programmes aimed at promoting the cessation of tobacco use, in such locations as: [sporting environments?]	design and implementation of programmes aimed at promoting the cessation of tobacco use, in such locations as: [other (please specify)?]	inclusion of diagnosis and treatment of tobacco dependence and counselling services for cessation of tobacco use in national programmes, plans and strategies for: [tobacco control?]	inclusion of diagnosis and treatment of tobacco dependence and counselling services for cessation of tobacco use in national programmes, plans and strategies for: [health?]	inclusion of diagnosis and treatment of tobacco dependence and counselling services for cessation of tobacco use in national programmes, plans and strategies for: [education?]	inclusion of programmes on the diagnosis and treatment of tobacco dependence in your health-care system?	Which structures in your health-care system provide programmes for the diagnosis and treatment of tobacco dependence? [primary health care]	Which structures in your health-care system provide programmes for the diagnosis and treatment of tobacco dependence? [secondary and tertiary health care]	Which structures in your health-care system provide programmes for the diagnosis and treatment of tobacco dependence? [specialist health-care systems (please specify below)]	Which structures in your health-care system provide programmes for the diagnosis and treatment of tobacco dependence? [specialized centres for cessation counselling and treatment of tobacco dependence]	Which structures in your health-care system provide programmes for the diagnosis and treatment of tobacco dependence? [rehabilitation centres]
1	1	0	1	0	1	1	0	0	0	1	1	1	1	1	0	0	0	0
1	1	0	1	0	1	1	0	1	0	1	1	0	1	1	0	0	0	0
1	1	1	1	0	1	1	1	0	0	0	1	0	1	1	1	0	1	1
1	1	0	1	0	0	1	0	0	1	1	0	0	0	0	0	0	0	0
1	1	1	1	0	1	1	1	1	0	1	1	0	1	0	0	0	1	0
1	1	1	1	1	1	1	1	1	1	1	1	1	1	1	1	1	1	0
1	0	0	1	0	0	1	0	0	1	0	0	0	1	1	0	0	0	0
1	0	1	1	0	1	1	1	1	0	1	1	1	1	1	0	0	1	0
0	0	1	1	1	1	1	1	0	0	1	1	1	1	1	1	0	0	0
1	1	0	1	1	1	1	1	0	1	1	1	1	1	1	1	0	1	0
1	1	1	1	1	1	1	1	0	0	1	1	0	1	1	1	1	1	0
1	0	1	1	1	1	1	1	0	0	1	1	1	1	1	1	1	0	0
1	1	0	1	0	0	1	0	0	0	1	1	1	1	1	1	0	1	0
1	1	1	1	0	1	1	1	1	0	1	1	1	1	1	1	1	1	0
1	1	1	1	1	0	1	1	0	0	1	1	0	1	1	1	1	1	0
1	1	1	1	0	0	1	1	0	0	1	0	0	1	1	0	0	0	0
1	1	1	0	0	1	1	1	0	0	1	1	0	1	1	1	0	1	1
1	1	0	1	0	1	1	0	0	0	1	1	1	1	1	1	1	1	0
1	0	1	1	0	1	1	1	0	0	1	1	1	1	1	1	1	0	1
1	1	1	1	0	1	1	1	1	0	1	0	1	0	0	0	0	0	0
1	1	1	1	1	1	1	1	1	0	1	1	1	1	1	1	1	1	1
1	1	1	1	1	1	1	0	0	1	1	1	1	1	1	0	1	0	0
1	1	1	1	1	1	1	1	0	1	1	1	0	1	1	0	0	1	0
0	1	0	1	0	1	1	1	1	0	1	1	1	1	1	0	1	1	0
1	1	1	1	0	1	1	1	1	0	1	1	0	1	1	0	1	1	0
1	1	1	1	1	0	0	0	0	1	0	1	0	1	1	1	0	0	1
0	0	0	0	0	0	0	0	0	0	0	0	0	0	0	0	0	0	0
1	1	1	1	1	0	1	1	0	1	1	1	1	1	1	1	0	1	0
1	1	1	1	0	1	1	1	0	0	1	1	1	1	1	1	0	0	0
1	1	0	1	0	0	1	0	0	0	1	1	1	0	0	0	0	0	0
1	1	1	1	0	1	1	1	1	0	1	1	1	1	1	1	1	1	1
1	1	0	1	1	0	1	0	0	0	1	1	1	1	1	1	1	1	1
1	1	1	1	0	1	1	1	0	0	1	1	1	1	1	1	1	1	0
1	1	0	1	0	0	1	1	0	0	1	1	0	1	1	1	0	0	1
1	1	1	1	0	0	1	0	0	0	1	1	0	1	1	0	0	1	0
1	1	1	1	0	0	1	0	0	0	1	0	0	1	1	1	1	1	0
1	1	1	1	0	0	0	0	0	0	1	1	0	1	1	0	0	0	0
0	0	0	0	0	0	0	0	0	0	0	0	0	1	0	0	0	0	0
1	1	0	1	1	1	1	0	0	1	1	1	0	1	1	1	1	1	0
1	1	1	1	0	1	1	0	0	0	1	1	0	1	0	0	1	1	0
1	1	1	1	0	1	1	1	0	0	1	1	0	1	1	0	0	1	0
1	1	1	1	1	1	1	1	1	1	0	1	1	1	1	1	1	1	1
1	0	1	0	0	1	1	1	0	0	1	1	0	1	1	1	1	1	0
0	1	0	1	0	1	1	1	1	0	1	1	1	0	0	0	0	0	0
1	1	0	1	0	1	1	1	0	0	1	1	1	1	0	1	1	1	0
1	1	1	1	0	1	1	1	1	0	1	1	1	1	1	1	0	1	0
1	1	1	1	0	1	1	1	1	0	1	1	0	1	0	0	1	1	1
1	0	0	0	0	0	0	0	0	0	0	0	0	0	0	0	0	0	0
1	1	1	1	1	1	1	1	1	0	1	1	1	1	1	1	1	1	1
1	0	0	1	0	0	1	0	0	0	0	1	0	1	1	1	0	0	0

Are the services provided in these settings covered by public funding or reimbursement schemes? [primary health care]	Are the services provided in these settings covered by public funding or reimbursement schemes? [secondary and tertiary health care]	Are the services provided in these settings covered by public funding or reimbursement schemes? [specialist health-care systems (please specify below)]	Are the services provided in these settings covered by public funding or reimbursement schemes? [specialized centres for cessation counselling and treatment of tobacco dependence]	Are the services provided in these settings covered by public funding or reimbursement schemes? [rehabilitation centres]	Are the services provided in these settings covered by public funding or reimbursement schemes? [other (please specify below)]	facilitating accessibility and/or affordability of pharmaceutical products for the treatment of tobacco dependence?	Which pharmaceutical products are legally available for the treatment of tobacco dependence in your jurisdiction? [nicotine replacement therapy]	Which pharmaceutical products are legally available for the treatment of tobacco dependence in your jurisdiction? [bupropion]	Which pharmaceutical products are legally available for the treatment of tobacco dependence in your jurisdiction? [varenicline]	Which pharmaceutical products are legally available for the treatment of tobacco dependence in your jurisdiction? [other (please specify)]	Are the costs of these products covered by public funding or reimbursement? [nicotine replacement therapy]	Are the costs of these products covered by public funding or reimbursement? [bupropion]	Are the costs of these products covered by public funding or reimbursement? [varenicline]	Are the costs of these products covered by public funding or reimbursement? [other (please specify below)]
1	0	0	0	0	0	0	0	0	0	0	0	0	0	0
1	0	0	0	0	0	1	1	0	0	1	0	0	0	0
0.5	0.5	0	0.5	1	0	1	1	0	1	1	0.5	0.5	0.5	0
0	0	0	0	0	0	0	0	0	0	0	0	0	0	0
0.5	0.5	0.5	0.5	0.5	0	1	1	0	0	0	0	0	0	0
0.5	0.5	0.5	0.5	0.5	0	1	1	1	1	0	0	0.5	0.5	0
1	0	0	0	0	0	1	1	1	1	0	0	0	0	0
0.5	0	0	1	0	1	0	0	0	0	0	0	0	0	0
1	1	1	0	0	1	1	1	1	1	0	0	0	0	0
1	1	0	1	0	1	1	1	1	1	0	1	0	0	0
0.5	0.5	0	0.5	0	0	1	1	1	1	0	0.5	0.5	0.5	0
1	1	1	1	1	1	1	1	1	1	0	0.5	0.5	0.5	0
1	1	0	1	0	0	1	1	1	1	0	0	0	0	0
0.5	0.5	0.5	0.5	0	0	1	1	1	1	1	0	0	0.5	0
0.5	0.5	0.5	0.5	0	0.5	1	1	1	1	0	0.5	0	0.5	0
0.5	0	0	0	0	0	0	0	0	0	0	0	0	0	0
0.5	0.5	0	0.5	0.5	0	1	0	0	0	0	0	0	0	0
0.5	0.5	0.5	0.5	0.5	0.5	0	0	0	0	0	0	0	0	0
0.5	0.5	0.5	0	0.5	0	1	1	0	1	0	0	0	0	0
0	0	0	0	0	0	1	1	1	1	0	0	0	0	0
1	1	1	1	1	0	1	1	1	1	0	0.5	0.5	0.5	0
1	0	1	0	0	0	1	1	1	1	0	0.5	0.5	0.5	0
1	0	0	0.5	0	0	1	1	1	1	1	0	0	0	0
0	0	0	0	0	0	1	0	0	0	0	0	0	0	0
0.5	0	0.5	0.5	0	0	1	0	0	1	0	0	0	0	0
1	1	0	0	1	0	1	1	0	1	1	0	0	0	0
0	0	0	0	0	0	0	0	0	0	0	0	0	0	0
0.5	0.5	0	0.5	0	0	1	1	1	1	0	0.5	0.5	0.5	0
1	1	1	0	0	0	1	1	1	1	0	0	0	0	0
0	0	0	0	0	0	0	0	0	0	0	0	0	0	0
1	0.5	0.5	0.5	0.5	0	1	1	1	1	1	0.5	0.5	0.5	0.5
0.5	1	1	0.5	1	0	1	1	1	1	0	0	0	0	0
1	1	1	1	0	0	1	1	1	1	0	0	0	0	0
1	1	0	0	0.5	0	1	1	1	1	1	0	0.5	0.5	0.5
1	0	0	0.5	0	0	0	0	0	0	0	0	0	0	0
0	0	0.5	0.5	0	0	1	1	1	1	0	0	0.5	0.5	0
1	0	0	0	0	0	0	0	0	0	0	0	0	0	0
0	0	0	0	0	0	1	0	0	0	0	0	0	0	0
1	1	1	1	0	0	1	1	1	0	1	0	0	0	0
0.5	0.5	0.5	1	0	0	1	1	0	1	0	0.5	0	0	0
1	0	0	1	0	0	1	1	0	1	0	0	0	0	0
1	1	1	0	0	0.5	1	1	1	1	1	0	0.5	0.5	0
0.5	0.5	0.5	0.5	0.5	0	1	1	1	1	0	0.5	0.5	0.5	0
0	0	0	0	0	0	0	0	0	0	0	0	0	0	0
1	1	1	1	0	0	1	1	0	0	1	0	0	0	0
1	1	0.5	0.5	0	0	1	1	1	1	0	1	1	1	0
1	1	1	1	1	0	1	1	1	1	0	0	0	0	0
0	0	0	0	0	0	1	1	0	1	1	0	0	0	0
1	1	1	1	1	1	1	1	1	1	0	1	1	1	0
0.5	0.5	0	0	0	0	0	0	0	0	0	0	0	0	0

	**National key regulations**					**Taxation and prices**						**Public awareness raising and communications**	**Cessation**
	smoking_bans_P	adherence_smokefree_comp_P	health_warn_W	bans_advertising_E	compliance_bans_adv_E	share_taxes_R	aff_cig_trend_R	aff_cig_trend_R_score	aff_cig_2000_R	aff_cig_2000_R_tert	aff_cig_2000_R_tert_score	antitobacco_mass_cam_W	help_quit_O
Albania	5		4	5		4	Yes	1	4.56%	HIGH	1.00	2	4
Andorra	3	7	2	2		5		0	1.00%	LOW	0.00	2	4
Armenia	4	8	5	4	8	3	No	-1	3.28%	HIGH	1.00	4	4
Austria	3	9	5	4	10	4	Yes	1	1.34%	LOW	0.00	4	5
Azerbaijan	3		3	5		3	No change	0	2.57%	MID	0.50	2	3
Belarus	2	6	5	4	8	4	Yes	1	0.88%	LOW	0.00	5	4
Belgium	2	8	5	4	10	5	Yes	1	1.77%	LOW	0.00	2	4
Bosnia and Herzegovina	2		2	4	8	5	Yes	1	6.09%	HIGH	1.00	4	4
Bulgaria	5	5	5	4	8	5	No	-1	2.97%	HIGH	1.00	2	4
Croatia	4		5	4		5	No change	0	2.75%	MID	0.50	2	4
Cyprus	4	7	5	4	10	4	Yes	1	1.96%	LOW	0.00	4	4
Czech Republic	3	9	5	4	10	5	Yes	1	2.07%	MID	0.50	4	5
Denmark	2	9	5	4		5	No change	0	1.59%	LOW	0.00	4	5
Estonia	2	10	5	4	10	5	No change	0	1.99%	LOW	0.00	5	4
Finland	2	10	5	5	10	5	Yes	1	2.04%	MID	0.50	4	4
France	3	8	5	4	10	5	Yes	1	2.91%	MID	0.50	5	4
Georgia	4	8	5	4	8	5	No change	0	3.71%	HIGH	1.00	5	4
Germany	2		5	4	10	4	Yes	1	1.76%	LOW	0.00	5	4
Greece	5		5	4		5	Yes	1	2.93%	MID	0.50	2	4
Hungary	4		5	4		4	Yes	1	3.20%	HIGH	1.00	2	4

Iceland	2	10	4	5	10	4	No change	0	1.86%	LOW	0.00	2	3
Ireland	5	10	5	4	10	5	No	-1	1.94%	LOW	0.00	5	5
Israel	3		4	2		5	Yes	1	2.36%	MID	0.50	2	4
Italy	2		5	4	8	5	Yes	1	2.20%	MID	0.50	2	4
Kazakhstan	4		5	4	8	4	Yes	1	1.22%	LOW	0.00	5	4
Kyrgyzstan	3	3	5	4	7	4	Yes	1	9.64%	HIGH	1.00	2	4
Latvia	4	8	5	4	10	5	No change	0	2.45%	MID	0.50	5	4
Lithuania	3	3	5	4	10	4	No change	0	2.39%	MID	0.50	2	4
Luxembourg	3		5	4		4	No change	0	0.56%	LOW	0.00	2	5
Malta	5		5	4		5	No	-1	2.21%	MID	0.50	2	4
Monaco	2		2	2		1		0			0.00	5	4
Montenegro	3		5	4		5	Yes	1	3.31%	HIGH	1.00	2	2
Netherlands	2		5	4	10	5	Yes	1	1.78%	LOW	0.00	2	5
Norway	5	10	4	4	7	4	Yes	1	2.02%	LOW	0.00	5	4
Poland	3	8	5	4	8	5	No change	0	2.75%	MID	0.50	3	4
Portugal	4	8	5	4		5	No change	0	2.33%	MID	0.50	2	4
Republic of Moldova	3		5	5	8	4	Yes	1	4.24%	HIGH	1.00	2	4
Romania	5	7	5	4	9	4	No change	0	3.79%	HIGH	1.00	2	4
Russian Federation	5	9	5	5		4	Yes	1	2.22%	MID	0.50	5	4
San Marino	3		2	4	8	1		0			0.00	2	2
Serbia	3	3	3	4	10	5	Yes	1	3.55%	HIGH	1.00	2	4
Slovakia	3	10	5	4		5	Yes	1	2.14%	MID	0.50	2	5
Slovenia	3		5	5	8	5	No change	0	1.73%	LOW	0.00	2	4
Spain	5	8	5	5	10	5	No change	0	2.13%	MID	0.50	4	4
Sweden	2		5	4		4	Yes	1	1.47%	LOW	0.00	4	5
Switzerland	2		4	2	10	4	Yes	1	1.15%	LOW	0.00	2	4
Tajikistan	5	3	5	4		3	No change	0	10.44%	HIGH	1.00	2	2

The former Yugoslav Republic of Macedonia	5		4	4	10	5	No change	0	2.93%	MID	0.50	2	4
Turkey	5	7	5	5	8	5	No change	0	3.47%	HIGH	1.00	5	5
Turkmenistan	5	9	5	4	10	3	Yes	1	10.62%	HIGH	1.00	5	4
Ukraine	4	8	5	4	9	4	Yes	1	4.40%	HIGH	1.00	5	3
United Kingdom	5	10	5	4	10	5	Yes	1	3.36%	HIGH	1.00	5	4
Uzbekistan	2		3	4		4	No change	0	4.82%	HIGH	1.00	2	4

© 2023 González-Marrón A. et al.
